# Lysine N-methyltransferase SETD7 promotes bladder cancer progression and immune escape via STAT3/PD-L1 cascade

**DOI:** 10.7150/ijbs.87182

**Published:** 2023-07-16

**Authors:** Jiancheng Lv, Qikai Wu, Kai Li, Kexin Bai, Hao Yu, Juntao Zhuang, Huanyou Sun, Haiwei Yang, Xiao Yang, Qiang Lu

**Affiliations:** Department of Urology, The First Affiliated Hospital of Nanjing Medical University, Nanjing 210029, China.

**Keywords:** SETD7, bladder cancer, proliferation, migration, immune escape

## Abstract

**Background:** The immunotherapy sensitivity of patients with bladder cancer (BCa) remains low. As the role of protein methylation in tumorigenesis and development becomes clearer, the role of lysine N-methyltransferase SET domain containing 7 (SETD7) in the progression and immune escape of BCa is worth studying.

**Methods:** The correlation between lysine methyltransferase family and prognosis or immunotheray sensitivity of BCa patients were analyzed, and SETD7 was screened out because of the significant correlation between its expression and survival data or immunotherapy sensitivity. The expression of SETD7 in BCa tissues and cell lines were explored. The functions of SETD7 were investigated by proliferation and migration assays. The role of SETD7 in BCa immune escape was validated by analyzing the correlation between SETD7 expression and tumor microenvironment (TME)-related indicators. The results were further confirmed by conducting BCa cell-CD8^+^ T cell co-culture assays and tumorigenesis experiment in human immune reconstitution NOG mice (HuNOG mice). Bioinformatic prediction, CO-IP, qRT-PCR, and western blot were used to validate the SETD7/STAT3/PD-L1 cascade.

**Results:** SETD7 was highly expressed in BCa, and it was positively associated with high histological grade and worse prognosis. SETD7 promoted the proliferation and migration of BCa cells. The results of bioinformatics, in vitro co-culture, and in vivo tumorigenesis assays showed that SETD7 could inhibit the chemotoxis and cytotoxicity of CD8^+^ T cells in BCa TME. Mechanistically, bioinformatics analysis, CO-IP assay, qRT-PCR, and western blot results indicated that SETD7 could increase the expression of PD-L1 via binding and promoting STAT3.

**Conclusions:** Taken together, SETD7 indicated poor prognosis and promoted the progression and immune escape of BCa cells. It has great potential to act as a new indicator for BCa diagnosis and treatment, especially immunotherapy.

## Introduction

As a highly prevalent malignant tumor in the urinary system^1^, bladder cancer (BCa) can be divided into muscle invasive (MIBC) and non-muscle invasive (NMIBC)^2^, ^3^. Considering the characteristics of high recurrence rate and easy metastasis, more effective diagnosis and treatment targets need to be determined for BCa^4^. In recent years, immunotherapy based on immunocheckpoint inhibitors (ICIs) have been verified to have good safety and efficacy in multiple cancer species^5^, ^6^. It also provides a new option for the comprehensive treatment of BCa. However, the overall response rate to immunotherapy in patients with BCa is less than 30%^7^. Therefore, new targets need to be developed to improve the efficiency of BCa treatment, especially immunotherapy.

Post-translational modifications (PTMs) of proteins play important roles in chromatin state and gene expression^8^, ^9^. These typical modifications, including methylation, phosphorylation, acetylation, and ubiquitination, could participate in the activation and silencing of genes^9^, ^10^. The different functions of these modifications depend on the site being modified^11^. Some proteins that participate in tumorgenesis and progression could be regulated by PTMs^12^, ^13^. As one of the most frequently modified amino acid among the 20 amino acids, lysine can be modified by acetyl, ubiquitinyl, glycosyl, methyl, and crotonyl^14^-^18^. Lysine methylation is a process that transfers three methyl groups to the ε-amine of lysine residue^19^. In recent years, an increasing number of studies has focused on lysine methylation, especially on its role in the development of tumor^17^, ^20^. For instance, the SETDB1-induced lysine methylation of AKT could promote the progression of non-small-cell lung carcinoma (NSCLC)^21^. EZH2-induced lysine methylation of TRβ could promote the proliferation and growth of hepatocellular carcinoma cells^22^. In addition, lysine methyltransferase EZH2 regulates the immune escape and immunotherapy of cancer cells by influencing the expression of PD-L1 or relative chemokines^23^, ^24^. Therefore, the relationship between lysine methylation and tumor immunity needs to be further explored.

SET domain containing 7 (SETD7), a lysine mono-methyltransferase, contains 366 amino acids^25^. Similar to other lysine methyltransferases, SETD7 has the SET domain, which is responsible for methylation catalysis^26^. SETD7 methylates over 30 proteins, which could participate in various cancer hallmarks^27^. SETD7 plays different roles in different tumors, and it can be either oncogene or tumor suppressor^28^. This phenomenon depends on the substrate that SETD7 modifies. For examples, SETD7 methylated K436 and K595 on GLI3, thus promoting the protein stability of GLI3 and the progression of NSCLC^29^. SETD7 was also reported to inhibit the progression of lung cancer via methylating the K140 of STAT3, thus inhibiting the activity and downstream target genes expression of STAT3^30^. In BCa, SETD7 was reported to be a tumor suppressor that could be regulated by the METTL3/YTHDF2 m6A pathway^31^. In the present study, SETD7 was upregulated in BCa tissues and cell lines. The expression of SETD7 was positively associated with poor prognosis in patients with BCa. It could promote the proliferation and migration of BCa cells. The bioinformatics analyses and immune function assays also indicated that SETD7 could promote the immune escape of BCa cells. SETD7 could increase the PD-L1 expression by binding and promoting STAT3. As reported, STAT3/PD-L1 pathway could promote the immune escape of colorectal cancer and T-cell lymphoma^32^, ^33^. In addition, STAT3 overexpression could decrease the anti-PD-L1 immunotherapy sensitivity in BCa^34^. The results of this study provided a new bio-target for BCa diagnosis and immunotherapy sensitization.

## Methods

### Analysis of prognostic data and immunotherapy response data of patients with BCa

Significant targets that are suggestive for the prognosis of patients with BCa were screed out by using Gepia (http://gepia.cancer-pku.cn/index.html), which is based on TCGA-BLCA data to explore the correlations between 13 lysine methyltransferases and the prognosis of patients with BCa. BEST (https://rookieutopia.com/), which was based on GSE173839, was used to predict the relationship between the expression level of 12 lysine methyltransferases (the site does not include SETD8-related data) and immunotherapy responses in patients with BCa. The correlations between SETD7 and the survival data of BCa patients were also investigated through the BEST website which based on the GSE48276 and GSE69795 dataset. The correlation between BCa tumor grade (TCGA-BLCA) and SETD7 expression was investigated through BEST.

### Enrichment pathways analysis

TISCH2 website (http://tisch.comp-genomics.org/home/), which was based on TCGA data, was used to explore the correlations between SETD7 expression and survival of patients with 33 types of cancer. BEST website, which was based on TCGA and 11 GEO dataset (GSE14520, GSE19423, GSE31684, GSE37815, GSE39281, GSE48075, GSE48276, GSE52219, GSE69795, GSE70691, and GSE154261), was used to further explore the gene enrichment pathways associated with SETD7 expression. GSEA and KEGG analyses were used to investigate the hallmarks associated with SETD7.

### Human Protein Atlas (HPA)

The HPA website (https://www.proteinatlas.org/) was used to explore the protein level of SETD7 in BCa and normal urothelial tissue.

### Exploration of the correlation between SETD7 and characteristics of tumor micro-environment (TME) in BCa

The required data, including TCGA-BLCA and GSE48075, were collected and preprocessed. The RNA sequencing (RNA-seq) data (FPKM value) were downloaded and log2 transformed. TME immunological characteristics included the tumor infiltration of immune cells, the expression of effector genes of the tumor relative immune cells, the expression of immunomodulators, and the expression of inhibitory immune checkpoints. The correlation between these markers and SETD7 expression levels was explored in the two sets of preprocessed data. The counts of infiltrated immune cells, immunomodulators, immune effector genes, and inhibitory immune checkpoints in different groups of GSE48075 and TCGA-BLCA are shown in Table S1-3.

### Patient samples and cell lines

All 40 pairs of BCa tissues were obtained from patients who received radical surgery from 2016 to 2021 at the First Affiliated Hospital of Nanjing Medical University. The tissues from BCa patients that were used in this study were approved by the Ethics Committee of The First Affiliated Hospital of Nanjing Medical University. All patients signed the informed consent. We confirmed the tissue as BCa tumor tissue through pathologically diagnosed, while the adjacent normal tissue was diagnosed by pathologically confirming without tumor tissue in the same patient. The BCa cell lines (T24, RT4, BIU87, UMUC3, 253J, J82 and 5637) and urothelial cell line (SV-HUC) were obtained from the Type Culture Collection of the Chinese Academy of Sciences (Shanghai, China).

### Immunohistochemistry

The paraffin-embedded BCa tumors were sliced into 4-mm slides. The tissue slides were rehydrated by dealing with different grades of ethanol. Then, the antigens were isolated using a microwave. The slides were dipped with 3% H_2_O_2_. Then, the slides were incubated with the SETD7 antibody (Abcam, USA) at 4 °C overnight. Afterward, the slides were treated with the HRP-conjugated antibody (Protech, USA). The pictures were observed and collected using a microscope. The positive rate of SETD7 was identified by at least two pathologists.

### Cell culture and transfection

The DMEM medium (Gibco, USA) with 10% fetal bovine serum (BI, Israel) was used to culture the T24 and UMUC3 cells. The cells were cultured in a 37 °C constant-temperature incubator with 5% CO2.

SETD7 overexpression plasmid and control vector were purchased from GenePharma (GenePharma, Shanghai, China). The UMUC3 and T24 cells were transfected with these media when growing to 50% confluence in six-well plates by using the Lipofectamine 3000 kit (Invitrogen, USA).

### RNA isolation and quantitative real-time PCR (qRT-PCR)

The TRIzol reagent (Invitrogen, USA) was used to extract RNAs from BCa tissues and cells. Afterward, RNAs were reversely transcribed into cDNAs by using HiScript II (Vazyme, China). QRT-PCR assays were performed using the StepOne Plus real-time PCR system (Applied Biosystems, USA). β-actin was used as internal control. The target RNA CT values were normalized through subtracting the β-Actin CT values. The primers were obtained from TsingKe (TsingKe, Nanjing, China), and they are listed in Table S4.

### Protein extraction and Western blot

The tissues or cells were lysed using the RIPA buffer (Sigma, USA). The concentrations of protein extractions were quantified by bicinchoninic acid (BCA) assays (Beyotime, China). The proteins were isolated and transferred to polyvinylidene fluoride (PVDF) membrane (Millipore, USA) by SDS-PAGE. The PVDF membranes were incubated with primary (1:1000, Cell Signaling and Technology, USA) and secondary antibodies (1:1000, Protech, China) after blocking with 5% skim milk. The protein levels were evaluated using Chemiluminescence (Bio-Rad, USA) and Image Lab Software.

### Cell proliferation assay

A total of 3,000 UMUC3 or 2,000 T24 cells were seeded in 96-well plates to study the role of SETD7 in tumor proliferation. The cell viability was determined using the cell counting kit-8 (CCK-8, Dojindo, Japan) every 24 hours (24, 48, 72, and 96 h). We discarded the original medium of the 96-well plates and then added with new medium containing 10ul CCK-8 reagent. After incubating for an hour, the absorbance values at 450 nm were detected using a microplate reader (Tecan, Switzerland).

### Cloning formation

A total of 1,000 T24 or 1,500 UMUC3 cells were seeded in six-well plates. After 2 weeks, the cell colonies were fixed and stained with 4% paraformaldehyde and 0.1% crystal violet consecutively. The cell colonies were fixed for 15 minutes by adding 1mL 4% paraformaldehyde. Then the colonies were washed for several times by using PBS. After that, we stained the cell colonies for 15 minutes by adding 1mL 0.1% crystal violet. After washing by PBS for several times again, the cell colonies were quantified using the Image J software.

### Scratch wound healing assay

The monolayer T24 or UMUC3 cells were scraped off with a 200 μL pipette tip after growing over 90% confluence in six-well plates. Then, the cells were cultured with serum-free medium. After 48 h, the images of BCa cells were obtained using a microscope (Olympus, Japan).

### Transwell assays

A total of 20,000 T24 or 30,000 UMUC3 cells were seeded in the upper chamber (Corning, USA) of the Transwell system. The upper chamber was added with serum-free medium, and the lower chamber was added with normal medium. The BCa cells of top chambers were fixed and stained with 4% paraformaldehyde and 0.5% crystal violet consecutively. The results were observed and collected using an Olympus microscope.

### Co-inmunoprecipitation (CO-IP)

CO-IP assay was performed by using IP/CO-IP Kit (Absin, China) according to the manufacturer's protocol. Immunoprecipitations of SETD7 and STAT3 were conducted using an anti-SETD7 antibody (1:120. Abcam, USA) overnight at 4 °C. Then anti-STAT3 (1:1000, Protech, USA) was used for further western blot analysis.

### CD8^+^ T cell culture and CD8^+^ T cell-mediated BCa cell killing assay

The peripheral blood mononuclear cells (PBMCs) in the peripheral blood of healthy person were screened using the PBMC separation reagent (FACs, Nanjing, China). Then, the CD8^+^ T cells were isolated from PBMCs by using CD8 microbeads (Miltenyi, Germany) and magnetic separation. Afterward, the isolated CD8^+^ T cells in RPMI-1640 medium were cultured and activated by adding interleukin 2 (IL-2, 5 ng/mL; R&D Systems, USA), CD3 antibodies (2 μg/mL; Invitrogen, USA), and CD28 antibodies (1μg/mL; Invitrogen, USA). Then, the activated CD8^+^ T and BCa cells were co-cultured at a ratio of 1:2.5 for 48 h. Afterward, a proper amount of medium was collected from the co-culture system to detect the amount of granzyme B and IFN-γ by using the ELISA kit (FACs, Nanjing, China). The killing ability of CD8^+^ T cells was studied by co-culturing the activated CD8^+^ T cells with BCa cells at a ratio of 2:1 for 72 h. After removing the CD8^+^ T cells and debris by washing with PBS for several times, the amount of living BCa cells was detected using a spectrometer at 570 OD. Then, the living BCa cells were fixed and stained with 4% paraformaldehyde and 0.1% crystal violet consecutively. The process of CD8^+^ T cell screening and co-culture system construction has been reported in our previous study^35^.

### Chemotaxis Assay

A chemotaxis assay system was established by placing a 3 μm core diameter (Corning, USA) into a 24-well Transwell plate. Approximately 2 × 10^5^ activated CD8^+^ T cells were added into the upper chamber, and 1 × 10^6^ BCa cells were added into the lower chamber. CD8^+^ T cells migrated into the lower chamber were collected and counted by CCK-8 technique after 24 h of incubation.

### Humanized NOG mice generation and tumorgenesis model

NOG (NOD-scid IL2Rgnull) mice were obtained from Shanghai Charles River Co. NOG mice were severely deficiency in immune ability. The function of T cells, B cells, and NK cells in NOG mice were lost, and the function of macrophage was decreased. 10^7^ PBMCs screened from healthy human peripheral blood were transfecting into the NOG mice (5 weeks old, female) via tail vein. At the same time, T24 cells (n=10^7^) were injected into the axilla of NOG mice for tumorgenesis. Five days after the tumor xenograft, the tumor size was measured every 3 days. All human immune reconstitution NOG mice (HuNOG) were euthanized after 4 weeks, and the tumor samples were removed for further measurement and immunohistochemical testing. SETD7 (1:200, Protech, China) and CD8 primary antibodies (1:10000, Protech, China) were used for specific binding. Then HRP-conjugated antibody (1:5000, Abcam, USA) was used for for further luminescence detection. The Ethical approval of animal assays were obtained from the Animal Ethics Board of Nanjing Medical University.

### Statistical analysis

The data were analyzed using SPSS version 22.0 (IBM Corp., Armonk, NY, USA), and the results are expressed as mean ± standard deviation (means ± SD). Student's t-test and one-way ANOVA were used to investigate the differences between different groups. The overall survival (OS) was analyzed using the Kaplan-Meier method. The results were statistically significant when P values were less than 0.05.

## Results

### High expression of SETD7 suggests poor prognosis and poor immunotherapy response in patients with BCa

The Gepia database was used to investigate the correlation between 13 lysine methytransferase and OS or disease-free survival (DFS) of patients with BCa (Figure S1). The results showed high expression of EZH2 (pHR=0.035), and SETD7 (pHR=0.013) indicated poor DFS in BCa (Figure S1). Moreover, the high expression of SETD7 (pHR=0.065) suggests a trend of poor OS in patients with BCa (Figure S1). The BEST database was used to investigate the correlation between 12 lysine methytransferase (SETD8 was not included in BEST database) and immunotherapy response of patients with BCa in GSE173839 (Figure S2). The results showed that the high expression of SETDB1 (p=0.02) and EZH2 (p=0.019) indicated good response of anti-PD-L1 immunotherapy in patients with BCa (Figure S2). The high expression of SMYD3 (p=0.011) and SETD7 (p=0.0042) indicated no response of anti-PD-L1 immunotherapy in patients with BCa (Figure S2). Considering that SETD7 is associated with both prognosis and immunotherapy sensitivity in patients with BCa, its role in BCa progression and immune escape was further explored.

### SETD7 was upregulated in BCa tissues and cell lines and associated with poor prognosis

**Figure 1:**
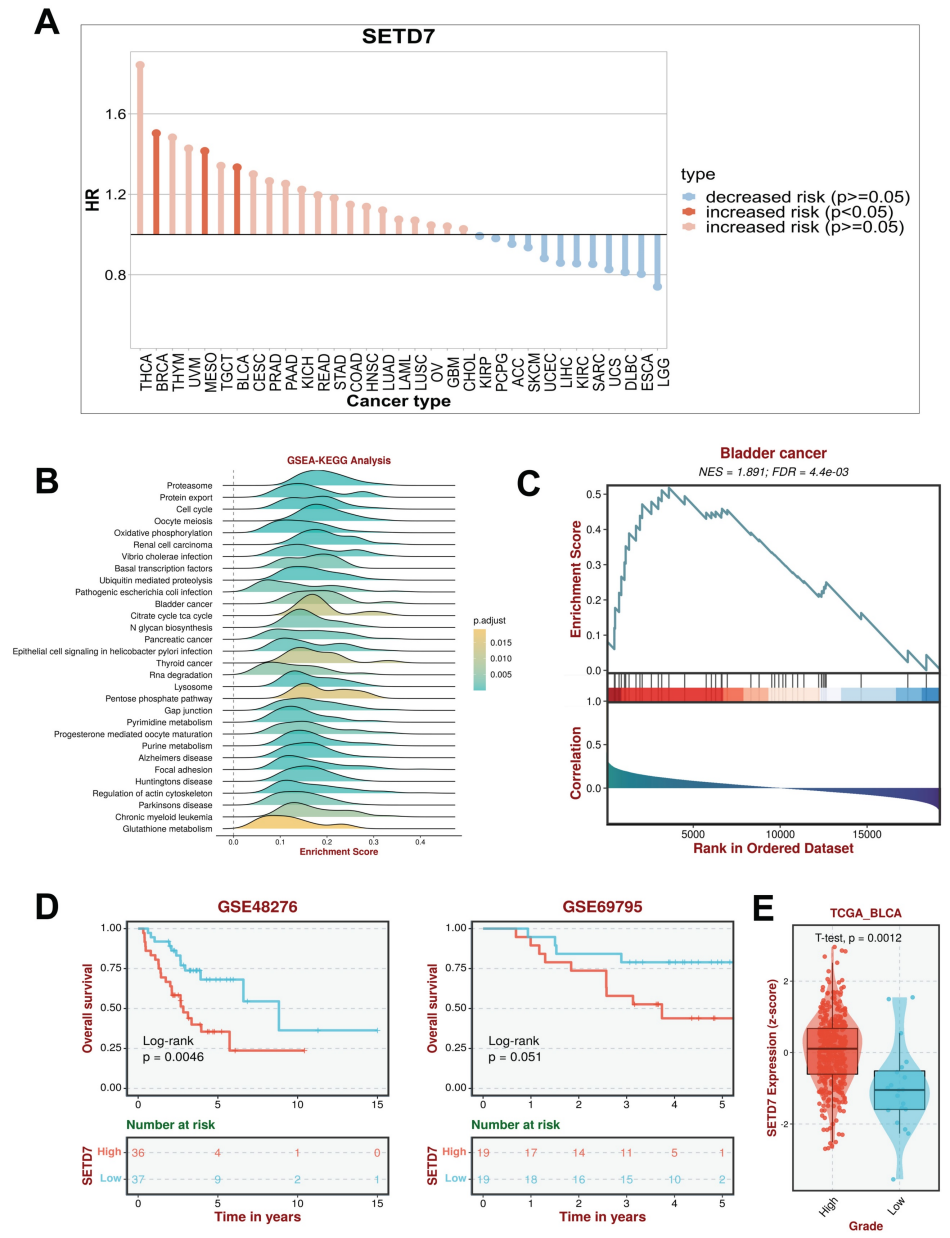
** SETD7 acted as a high-risk factor for BCa progression and worse survival.** SETD7 was an increased risk factor in BRCA, MESO, and BLCA. **B.** Gene enrichment analysis result showed that SETD7 was correlated with 'bladder cancer' pathway.** C.** GSEA analysis showed bladder cancer associated genes were influenced by SETD7.** D.** GSE48276 and GSE69795 data indicated that SETD7 was correlated with worse overall survival of BCa patients. **E.** TCGA-BLCA data indicated that SETD7 was highly regulated in high-grade BCa patients compared with low-grade patients.

**Figure 2:**
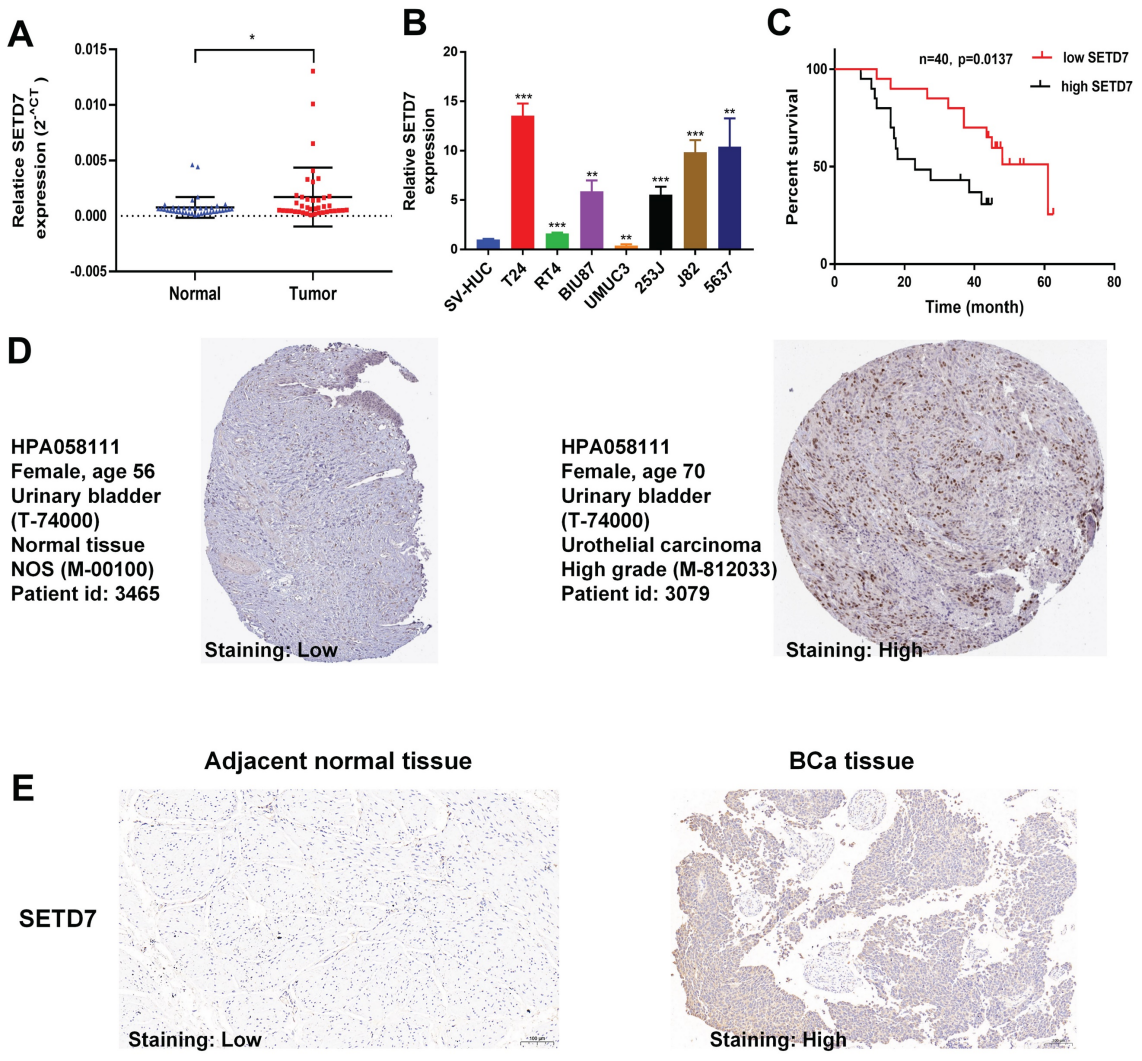
** SETD7 was highly regulated in BCa.** The expression of SETD7 in 40 pairs of BCa tissues was validated by qRT-PCR (*P<0.05, Student's t-test). **B.** The expression of SETD7 in seven BCa cell lines and SV-HUC cell line was confirmed by qRT-PCR (**P<0.01, ***P<0.001, Student's t-test). **C.** The correlation between SETD7 and the overall survival of patients with BCa was validated by Kaplan-Meier analysis. **D.** The expression of SETD7 in BCa and normal tissues was validated using the Human Protein Atlas (HPA) database. **E.** The expression of SETD7 in our own BCa tissues and adjacent normal tissues was confirmed by IHC. Data are expressed as mean±SD, n=3.

TISCH2 was used to explore the correlation between SETD7 and survival of patients with 33 types of cancer. The results indicated that SETD7 was an increased risk for breast cancer, mesothelioma and BCa (Figure 1A). The BEST database was used to further explore the gene enrichment pathways associated with SETD7 expression. According to the results of GSEA and KEGG analysis, SETD7 was positively associated with bladder cancer relative pathway (Figure 1B-C). The GO-GSEA and Hallmark-GSEA analyses were also performed. The result of GO-GSEA analysis showed that SETD7 was negatively associated with natural killer activation involved in immune response pathway (Figure S5A). The result of Hallmark-GSEA analysis showed that SETD7 was positively associated with inflammatory response pathway and complement pathway (Figure S5B). BEST database analysis (GSE48276 and GSE69795) also indicated that SETD7 was positively correlated with worse OS among patients with BCa (Figure 1D). According to the data of TCGA-BLCA, SETD7 was positively associated with a high pathological grade in BCa patients (Figure 1E). The expression of SETD7 was investigated in 40 pairs of BCa tissues, and the result showed SETD7 was highly regulated in BCa tissues compared with adjacent normal tissues (Figure 2A). In addition, SETD7 was highly expressed in 7 BCa cell lines compared with SV-HUC (Figure 2B). The high and low SETD7 expression samples were distinguished by the median value of 40 samples. The expression of SETD7 was positively correlated with tumor stage in patients with BCa (Table 1). Kaplan-Meier analysis indicated that patients with higher SETD7 expression had a poor overall survival (Figure 2C). According to the HPA database, SETD7 was more highly expressed in BCa tissues compared with normal bladder tissue (Figure 2D). Immunohistochemistry results showed that SETD7 was highly expressed in BCa tissues compared with adjacent normal tissue (Figure 2E).

### SETD7 promoted the proliferation and migration of BCa cells in vitro

**Figure 3:**
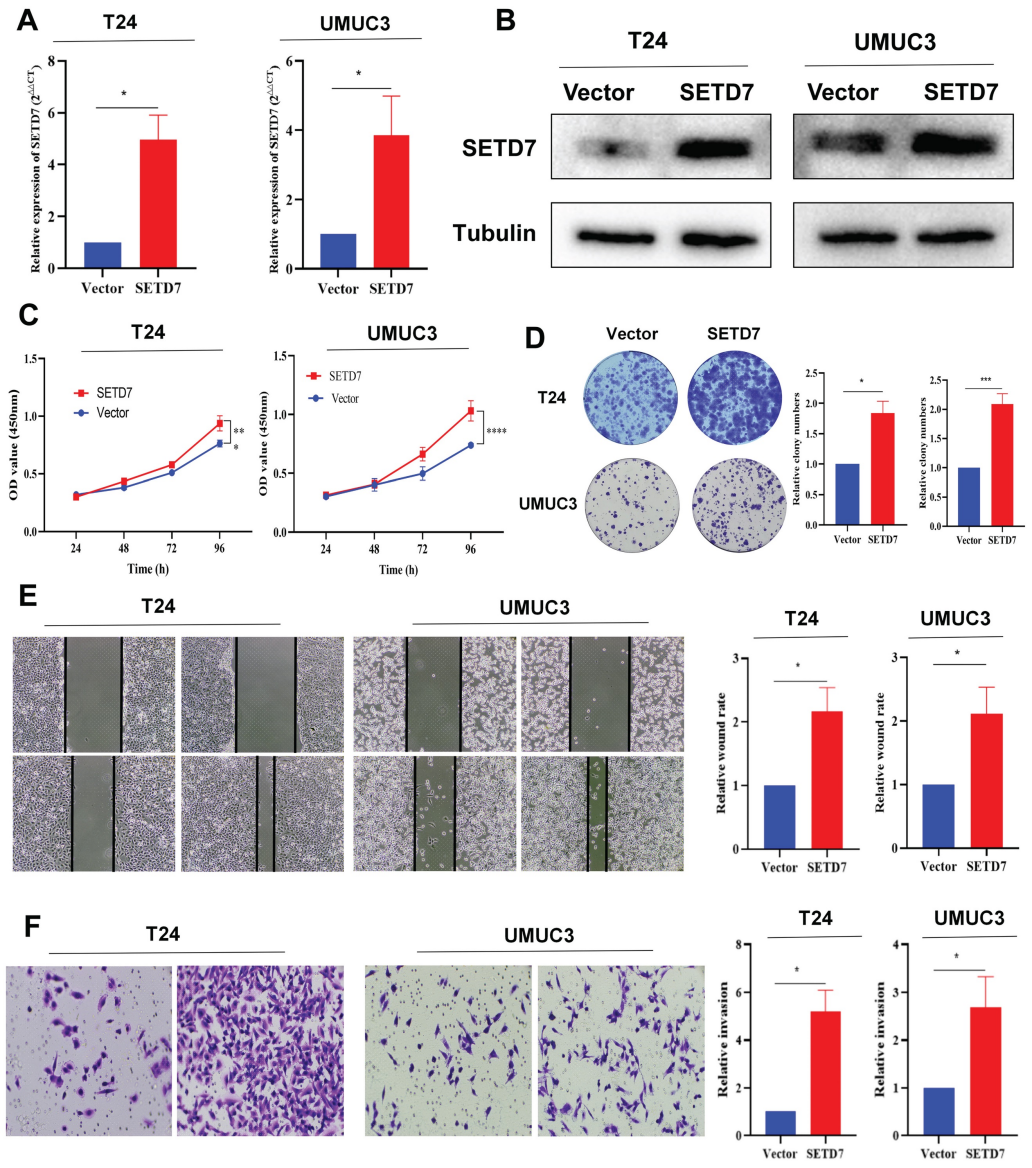
** SETD7 promoted the proliferation and migration of BCa cells. A.** Efficiency of SETD7 overexpression in T24 and UMUC3 cells was confirmed by qRT-PCR (*P<0.05, Student's t-test).** B.** Efficiency of SETD7 overexpression in T24 and UMUC3 cells was confirmed by Western blot analysis.** C.** CCK8 assay showed that SETD7 could promote T24 and UMUC3 cell proliferation (***P<0.001, ****P<0.0001, Student's t-test).** D.** Colony formation assay indicated that SETD7 could promote T24 and UMUC3 colony formation (*P<0.05, ***P<0.001, Student's t-test).** E.** Scratch wound healing assays showed that SETD7 could promote the migration rate of T24 and UMUC3 cells (*P<0.05, Student's t-test).** F.** Transwell assays showed that SETD7 could promote the migration rate of T24 and UMUC3 cells (*P<0.05, Student's t-test). Data are mean±SD, n=3.

We separately chose a high SETD7 expression BCa cell line T24 and low SETD7 expression BCa cell line UMUC3 for further experimental validation. The SETD7 overexpression plasmid and control vectors were transfected into the T24 and UMUC3 cells. The results of qRT-PCR and Western blot showed that SETD7 was overexpressed in T24 and UMUC3 cells after transfection (Figure 3A-B). The results of CCK-8 assay revealed that the SETD7 promoted the proliferation ability of T24 and UMUC3 cells (Figure 3C). Cloning formation assays also showed that SETD7 could increase the colony numbers of T24 and UMUC3 cells (Figure 3D). The results of scratch wound healing assays and transwell assays also revealed that SETD7 could promote the migration rate of UMUC3 and T24 cells (Figure 3E-F).

### SETD7 had a negative correlation with anti-tumor immune response of TME in BCa

**Figure 4:**
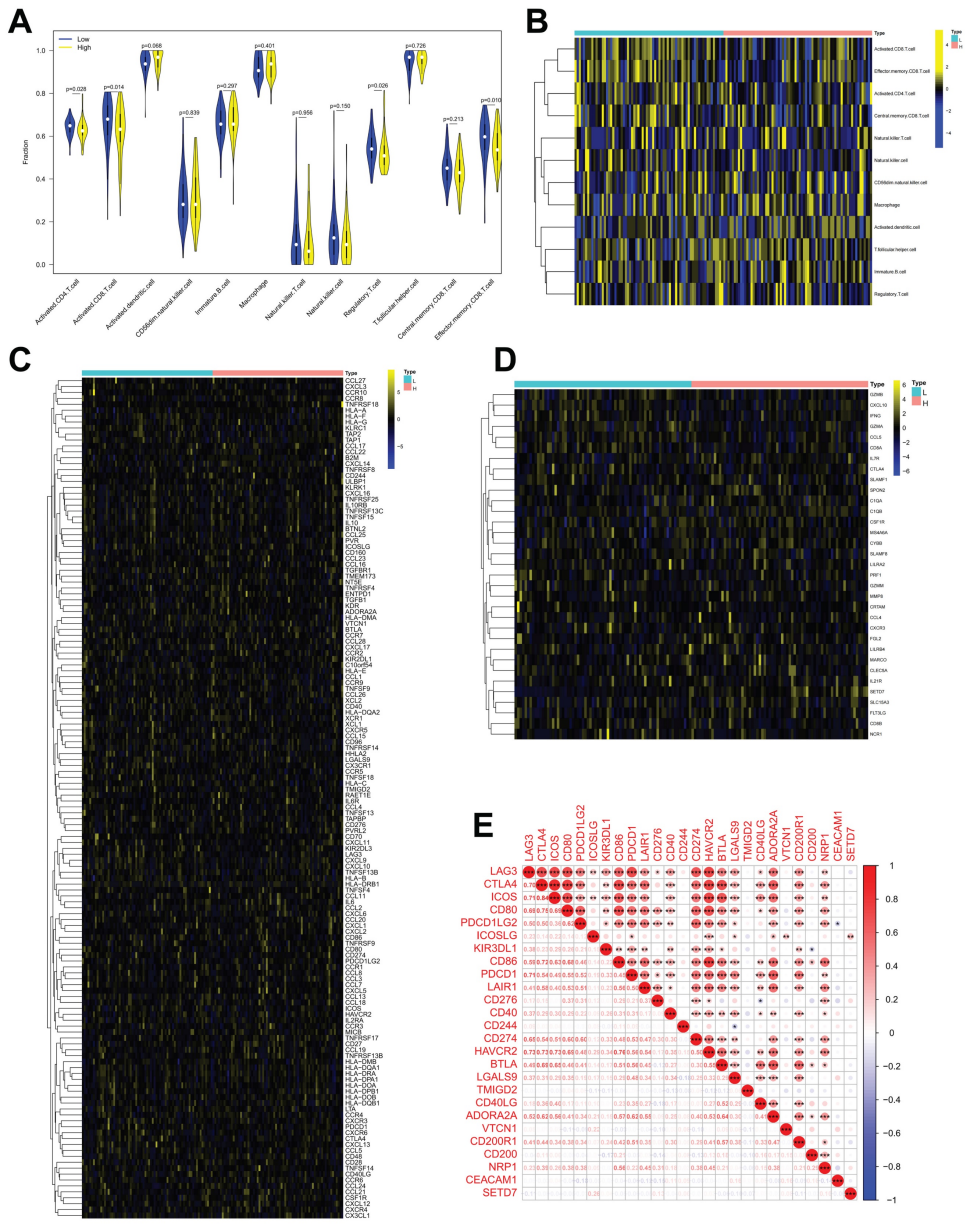
** SETD7 was positively associated with the immune escape ability of GSE48075. A-B.** Infiltration level of different immune cells in high- and low-SETD7 groups on the basis of GSE48075 data.** C.** Expression of different immunomodulators in high- and low-SETD7 groups based on the GSE48075 data. **D.** Expression of immune cells effector genes in high- and low-SETD7 groups on the basis of GSE48075 data.** E.** Correlation analysis between SETD7 and inhibitory immune checkpoints on the basis of GSE48075 data.

**Figure 5:**
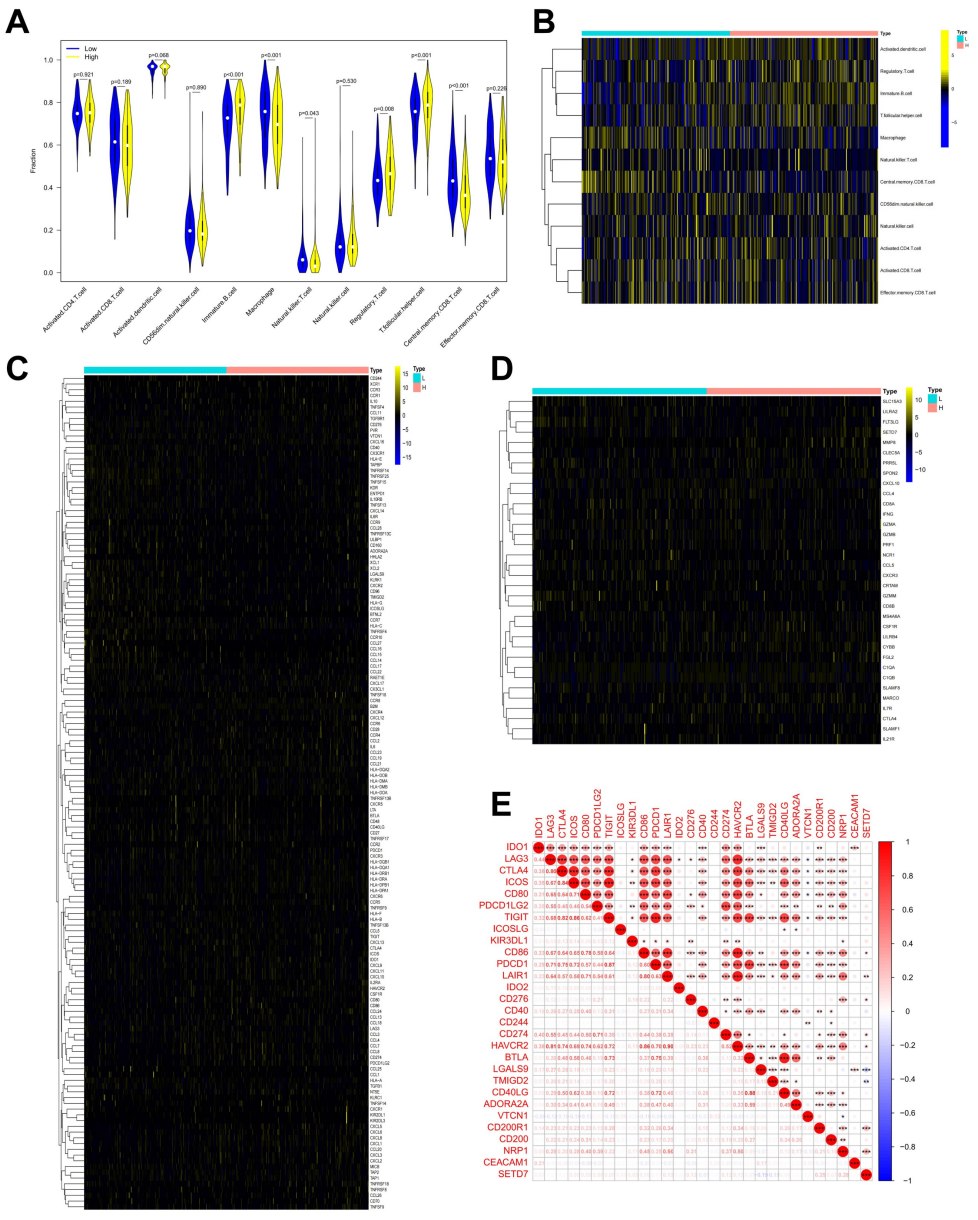
** SETD7 was positively associated with immune escape ability of BLCA. A-B.** Infiltration level of different immune cells in high and low SETD7 groups on the basis of TCGA-BLCA data.** C.** Expression of different immunomodulators in high- and low-SETD7 groups on the basis of TCGA-BLCA data. **D.** The expression of immune cells effector genes in high- and low-SETD7 groups on the basis of TCGA-BLCA data.** E.** Correlation analysis between SETD7 and inhibitory immune checkpoints on the basis of TCGA-BLCA data.

The role of SETD7 in immune response of BCa was explored by studying the correlations between SETD7 expression and infiltrated immune cells, immunomodulators, effector genes of immune, cells and inhibitory immune checkpoints in TME. According to the median expression value of SETD7, individuals were separated into high- and low-SETD7 group in GSE48075, TCGA-BLCA, and GSE69795 cohorts. The analysis results of GSE48075 showed that activated CD4 T cells, activated CD8 T cells, regulatory T cells, and effector memory CD8 T cells were less infiltrated in SETD7 highly expressed individuals (Figure 4A-B). Many immunomodulators, including LAG3, CXCL13, and CXCL5 were differentially expressed in high and low SETD7 groups of GSE48075 (Figure 4C, Table 2). The immune cells effector genes GZMA and GZMB were negatively correlated with SETD7 in GSE48075 (Figure 4D, Table 3). The inhibitory immune checkpoint ICOSLG was positively associated with SETD7 in GSE48075 (Figure 4E). According to the analysis results of TCGA-BLCA cohort, central memory CD8 T cells were less infiltrated in SETD7 highly expressed individuals (Figure 5A-B).

Many immunomodulators including TNFRSF4, TMIGD2, TNFRSF14, and TNFRSF25 were negatively associated with SETD7 in TCGA-BLCA cohort (Figure 5C, Table 4). Immune cell effector genes such as CYBB, FLT3LG, GZMM and SLC15A3 were differentially expressed in high or low SETD7 groups (Figure 5D, Table 5). The inhibitory immune checkpoints CD86, LAIR1, CD276, CD274 (PD-L1), HAVCR2, CD200R1, and NRP1 were positively associated with SETD7, while LGALS9 and YMIGD2 were negatively associated with SETD7 (Figure 5E). According to the analysis results of GSE69795, no particular type of infiltrated immune cells was associated with SETD7 (Figure S3A). While SETD7 had a trend to inhibit the immunescore which was based on whole infiltrated immune cells of GSE69795 (Figure S3B). Immunomodulators such as CD27, KDR, CCL27, CCR9, and CXCL17 were differentially expressed in high- or low-SETD7 groups (Figure S3C, Table S5). The immune cell effector genes GZMA, GZMB, and SLAMF1 were lowly expressed in high-SETD7 groups (Figure S3D, Table S6). The inhibitory immune checkpoints KIR3DL1 were positively associated with SETD7, while CTLA4, ICOS, IDO2, TMIGD2, and ADORA2A were negatively associated with SETD7 (Figure S3E). According to the whole analysis results of the three databases, SETD7 could inhibit the infiltration of immune cells such as CD8^+^ T cells and influence the expression of immunomodulators or effector genes such as PD-L1, GZMA, and GZMB. Therefore, a series of experiments was used to verify the role of SETD7 in the TME of BCa.

### SETD7 promoted PD-L1 expression via binding STAT3 in BCa cells

**Figure 6:**
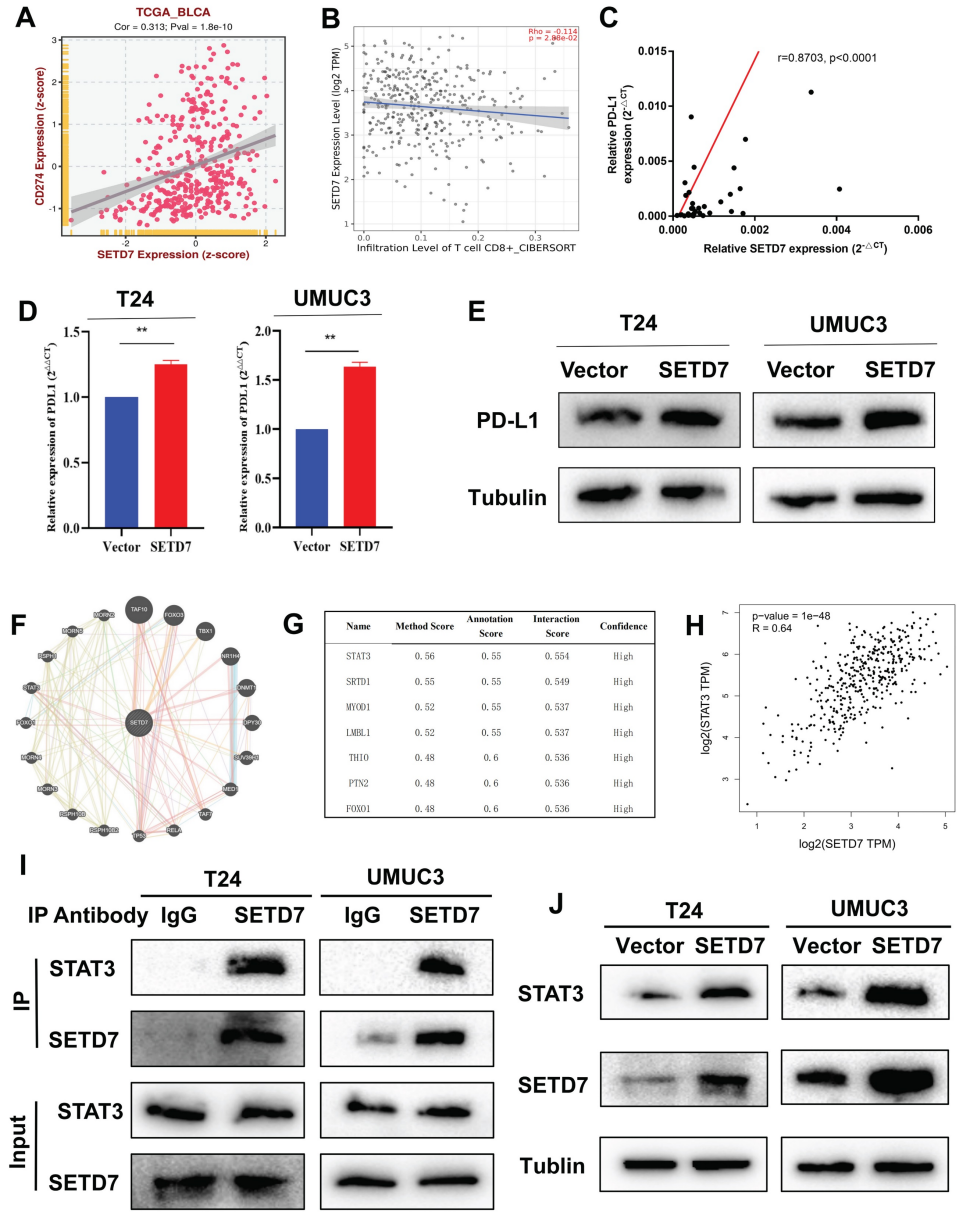
** SETD7 promoted the expression of PD-L1 in BCa. A.** Correlation analysis of SETD7 and PD-L1 in TCGA-BLCA.** B.** Correlation analysis of SETD7 and CD8^+^ T cell infiltration in BCa based on TCGA-BLCA.** C.** Pearson's correlation analysis was applied to confirm the correlation of SETD7 and PD-L1.** D.** QRT-PCR results revealed that SETD7 promoted the mRNA level of PD-L1 in T24 and UMUC3 cells (**P<0.01, Student's t test). **E.** Western blot results revealed that SETD7 promoted the protein level of PD-L1 in T24 and UMUC3 cells. **F.** Genemania database was used to predict the potential binding proteins of SETD7. **G.** Hitpredict database was used to predict the potential binding proteins of SETD7. **H.** Correlation analysis of SETD7 and STAT3 in TCGA-BLCA. **I.** CO-IP assay was used to confirm the binding of SETD7 and STAT3. **J.** Western blot assay indicated that SETD7 promoted the expression of STAT3. Data are expressed as mean±SD, n=3.

On the basis of TCGA-BLCA data, SETD7 was positively associated with the expression of PD-L1 and negatively with the CD8^+^ T cell infiltration (Figure 6A-B). In addition, SETD7 can also regulate the chemotaxis of other immune cells, such as inhibiting the infiltration of Treg cells, plasma cells and dendritic cells, and promoting the infiltration of neutrophils, mast cells and M1 macrophages (Figure S4A-F). The ESTIMATE algorithm was used to evaluate the role of SET7 on immune score and stromal score of TCGA-BLCA samples. The results showed SETD7 had a positive association with BCa immune score and stromal score (Figure S4G-H). The expression of PD-L1 in BCa tissues was positively associated with SETD7, as confirmed by Pearson's correlation analysis in 37 samples (Figure 6C). The results of qRT-PCR and Western blot showed that SETD7 overexpression could promote the expression of PD-L1 (Figure 6D-E). We predicted SETD7 binding proteins using Genemania (http://genemania.org/) and Hipredict (http://www.hitpredict.org/) websites (Figure 6F-G). As studies have reported that STAT3 could promote the expression of PD-L1, we screened it for further study. Correlation analysis confirmed SETD7 was positively associated with STAT3 in TCGA-BLCA (Figure 6H). CO-IP assays results confirmed SETD7 could bind STAT3 in T24 and UMUC3 cells (Figure 6I). Western-blot results showed SETD7 could promote the expression of STAT3 (Figure 6J).

### SETD7 inhibited the chemotaxis and function of CD8^+^ T cells in vitro

**Figure 7:**
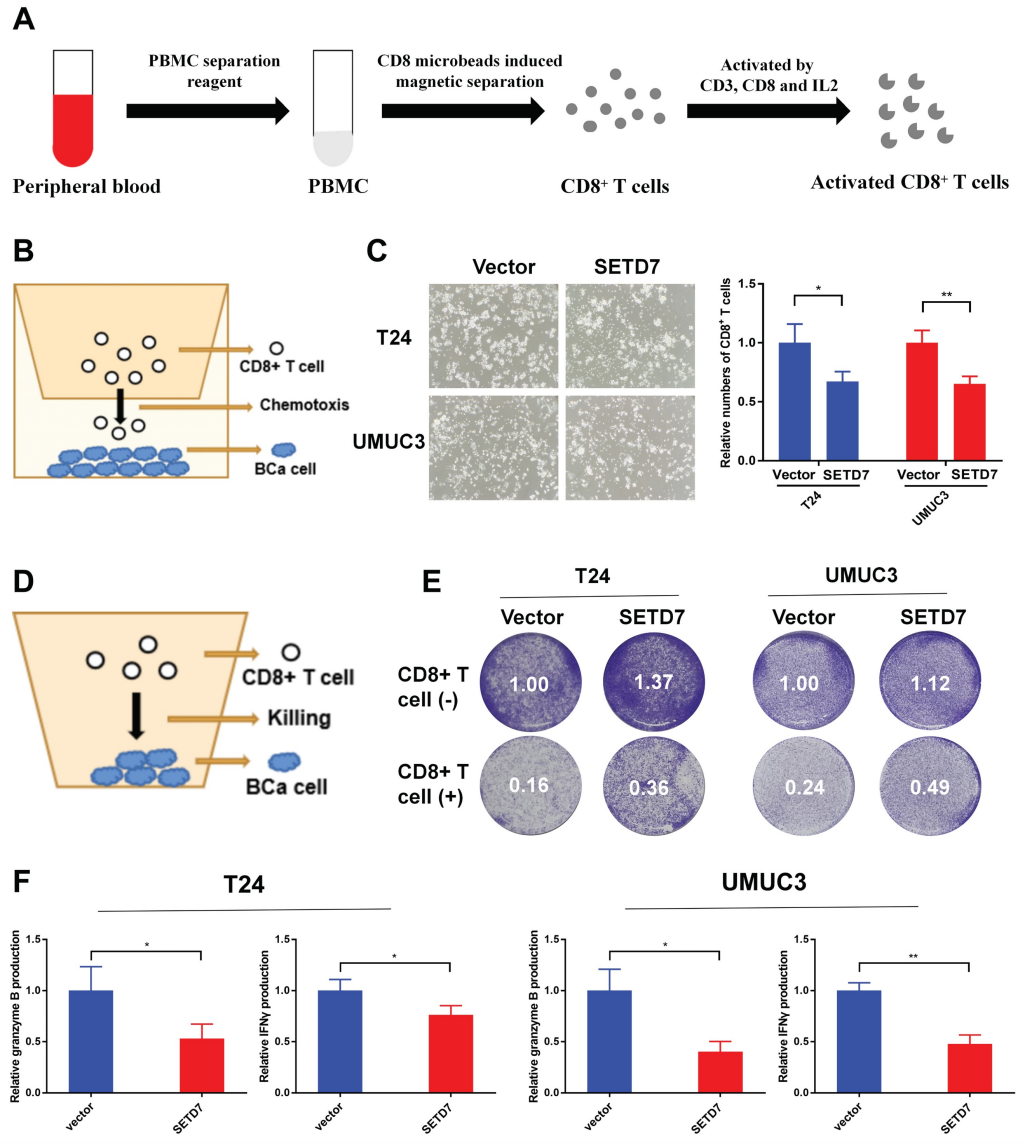
** SETD7 inhibited the chemotaxis and function of CD8^+^ T cells in vitro. A.** Schematic diagram of the process of the sorting and activatation of CD8^+^ T cells. **B.** Schematic diagram of CD8^+^ T cell chemotaxis assay. **C.** Chemotaxis assays results showed the migration ability of CD8^+^ T cells was decreased when co-culturing with SETD7 overexpression T24 or UMUC3 cells (*P<0.05, **P<0.01, Student's t-test). **D.** Schematic diagram of CD8^+^ T cell-mediated BCa cell killing assay. **E.** CD8^+^ T cell-mediated BCa killing assay results show that the killing ability of CD8^+^ T cell was decreased when co-culturing with SETD7 overexpression T24 or UMUC3 cells. **F.** ELISA assay results show that CD8^+^ T cells produced less IFN-γ and granzyme B when co-culturing with SETD7 overexpression T24 or UMUC3 cells (*P<0.05, **P<0.01, Student's t-test). Data are expressed as mean±SD, n=3.

The CD8^+^ T cells were screened and activated from the peripheral blood of healthy person (Figure 7A). The chemotaxis of CD8^+^ T cells was explored through a two-chamber co-culture model (Figure 7B). When co-culturing SETD7 overexpression T24 or UMUC3 cells with CD8^+^ T cells, less activated CD8^+^ T cells moved to the lower chamber (Figure 7C). The anti-tumor ability of CD8^+^ T cells were explored using a one-well co-culture model (Figure 7D). When co-culturing SETD7 overexpression T24 or UMUC3 cells with CD8^+^ T cells, the anti-tumur ability of CD8^+^ T cells was inhibited (Figure 7E). The results of ELISA assays indicated that CD8^+^ T cells produced less granzyme B and IFN-γ upon co-culturing with SETD7 overexpressed T24 or UMUC3 cells (Figure 7F). Hence, SETD7 could inhibit the chemotaxis and function of CD8^+^ T cells in TME of BCa.

### SETD7 promoted the tumorgenesis and inhibited the infiltration of CD8^+^ T cells in HuNOG mice

**Figure 8:**
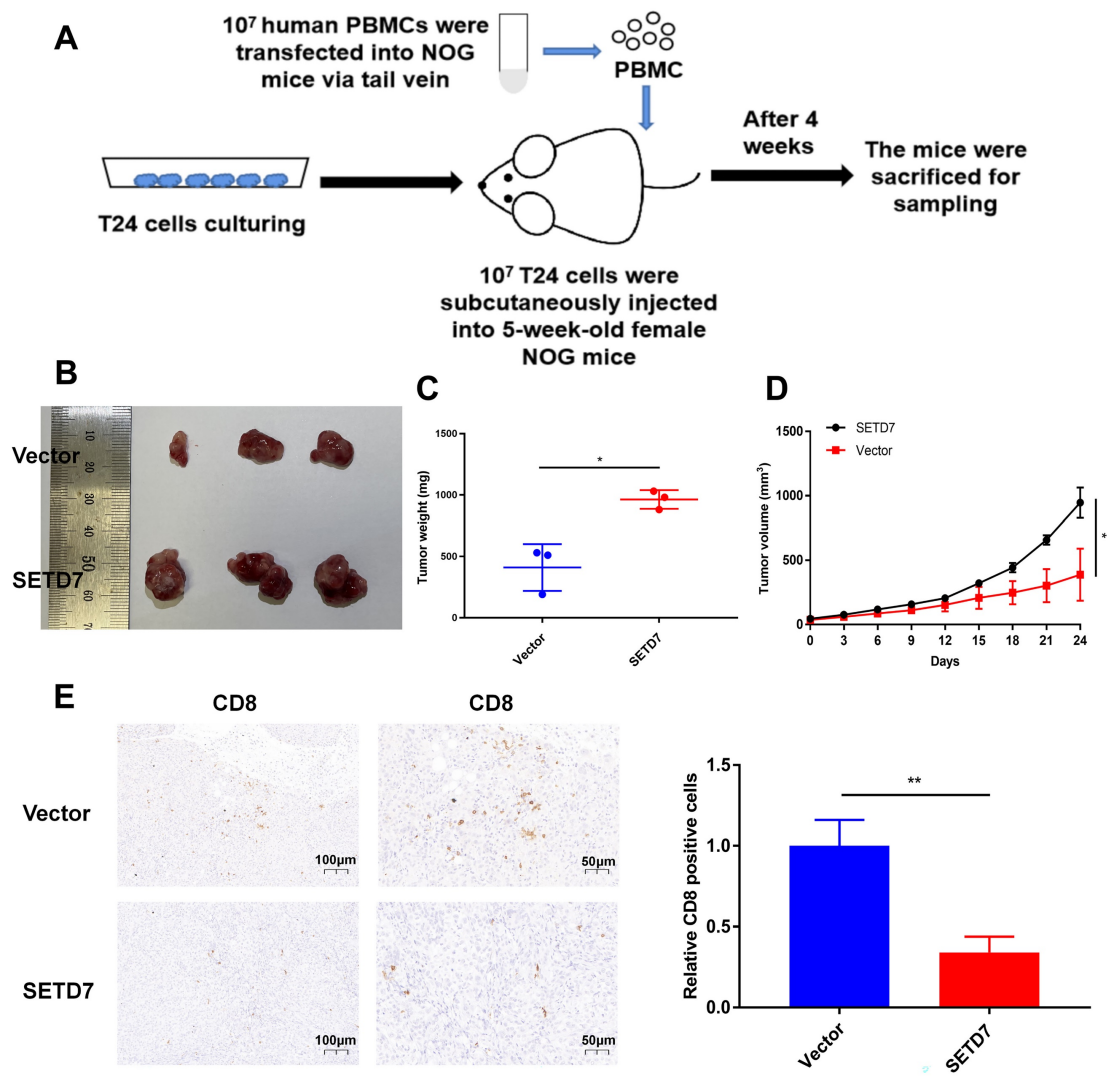
** SETD7 promoted the tumor proliferation and inhibited function of CD8^+^ T cells in vivo. A.** Schematic diagram of the HuNOG mice model construction.** B.** Representative picture of the tumor samples of HuNOG mice injected with SETD7 overexpression or control T24 cells (n=3). **C.** Weights of the tumor samples dissected from HuNOG mice (*P<0.05, Student's t-test). **D.** Tumor volumes of two groups of HuNOG mice were measured every 3 days (*P<0.05, Student's t-test). **E.** IHC staining results indicated SETD7 inhibited CD8 positive rate in tumor samples dissected from HuNOG mice (**P<0.01, Student's t-test). Data are expressed as mean±SD, n=3.

The role of SETD7 in BCa tumorgenesis and function of CD8^+^ T cells was explored by constructing HuNOG mice model (Figure 8A). The tumor samples were dissected from the HuNOG mice after 4 weeks of measurement (Figure 8B). The measurement results showed that SETD7 could increase the weight and volume of tumors in vivo (Figure 8C-D). IHC staining results showed SETD7 could inhibit the infiltration of CD8^+^ T cells in BCa tumors (Figure 8E).

## Discussion

In the present study, SETD7 was screened out from 13 major lysine methylation-associated proteins by comparing the prognosis and immunotherapy sensitivity data of BCa patients. The high expression of SETD7 in BCa tissues and cell lines were verified by bioinformatics analyses and experiments. The results confirmed that SETD7 was positively correlated with worse survival and higher tumor stage. In vitro studies indicated that SETD7 overexpression could promote the proliferation and migration of BCa cells. The results of bioinformatics analysis and immunological assays confirmed that SETD7 could inhibit the function of CD8^+^ T cells and increase the PD-L1 expression via binding STAT3. Accordingly, a novel biomarker that could induce the progression and immune escape of BCa was identified, and this biomarker may play an important role in predicting the susceptibility of patients with BCa to immunotherapy.

Dynamic covalent PTMs such as acetylation, methylation, ubiquitination, and phosphorylation could influence the expression and function of many proteins^36^. These non-histone proteins could participate in different physiological and pathological pathways such as tumorigenesis and progression^37^. Many important oncogenes and tumor suppressor genes have been affected by PTMs^13^, ^38^. For example, the action of PTEN can be regulated by phosphorylation and ubiquitination modification^39^, ^40^. Therefore, the in-depth understanding of PTMs is helpful to reveal the law of tumor progression and provide a new direction for tumor treatment. Among these PTMs, lysine methylation represents a complex and versatile type of modification. It may participate in the activation or inactivation of target proteins by modifying different lysine sites^41^. During this process, the function, stability, interactions, subcellular location, and structure of proteins could be influenced^42^. Recently, some some novel mechanism and therapy target in BCa management has been raised. For instance, ETV4 could promote BCa lymphangiogenesis and LN metastasis, and NAT10 could induce cisplatin chemoresistance in BCa^43^, ^44^. As one of the lysine methyltransferases, SETD7's role in tumors is of particular interest. SETD7 could promote the tumor progression by methylating SMAD7, YAP, AR, and GLI3^29^, ^45^-^47^. It could also inhibit the tumor development by methylating STAT3 or HIF-1α^30^, ^48^. However, limited studies have focused on SETD7 in BCa, which arouses our concern.

As an emerging therapeutic approach in recent years, immunotherapy based on immune checkpoint inhibitors has brought new hope for the integrated management of BCa. However, according to clinical data, the response rate of immunotherapy in patients with BCa is only approximately 20%^7^. As an important regulatory factor in tumor occurrence and development, the role of lysine methyltransferase represented by SETD7 in tumor immune escape and immunotherapy is worthy of further study. In terms of immune response regulation, it has been reported that SETD7 could promote Hepatitis C virus replication by attenuating IFN pathway^49^. Not only that, it was also reported that SETD7 regulated the activation of T cells by influencing the IL-2 production^50^. According to the results of bioinformatic analyses and related experiments, SETD7 could also regulate the chemotaxis of immune cells. According to the chemokines expression analyses, SETD7 had a negative regulatory effect on the expression of CXCL13, CCL15, CCL17, and other chemokines. This could be an important reason why SETD7 inhibited the chemotaxis of CD8^+^ T cells to the TME. Therefore, SETD7 has important potential in regulating tumor immunity. According to our exploration, SETD7 could promote the expression of PD-L1 in BCa cells and inhibit the chemotaxis and function of CD8^+^ T cells in the local TME. As an important immune checkpoint, the high expression of PD-L1 not only promotes the immune escape of tumor cells and weakens the role of CD8^+^ T cells in the TME, but also has an important impact on the sensitivity of immunotherapy. The current findings show that SETD7 could increase the expression of PD-L1 via binding and promoting STAT3, which has been reported to promote PD-L1 in BCa^51^, ^52^. As reported, PD-L1 could not only promote BCa immune escape, but also promote the progression of BCa cells^53^. Not only that, SETD7 may regulate the chemotaxis and function of CD8^+^ T cells in many other ways, such as influencing the chemokines in TME. These factors depend on our further exploration of potential mechanism of SETD7 in the progression and treatment of BCa.

## Conclusion

**Figure 9:**
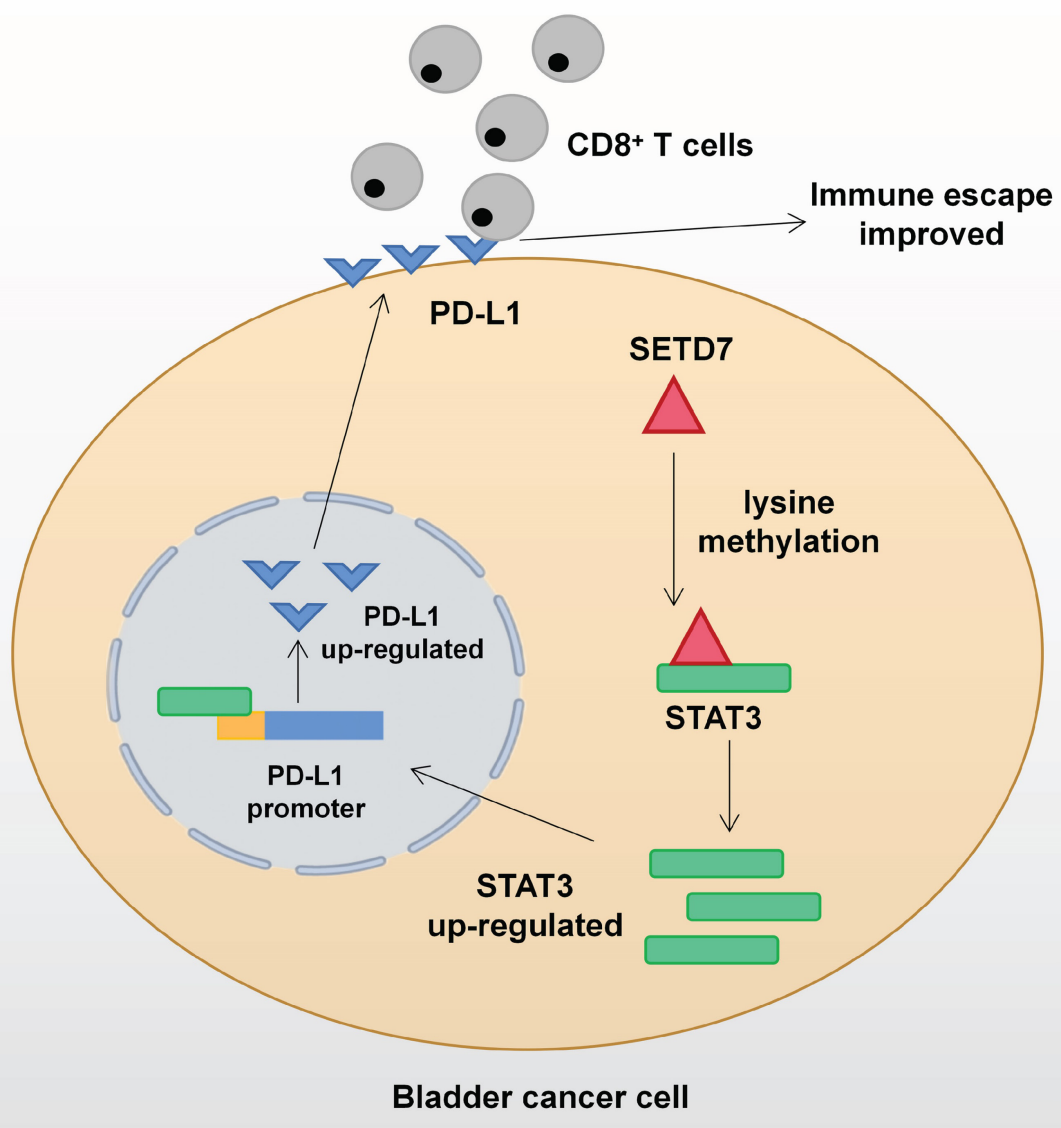
** Mechanism network of SETD7 in bladder cancer immune escape**.

The function of SETD7 as an oncogene in BCa has been demonstrated for the first time. SETD7 could promote the progression and immune escape of BCa. It was positively associated with worse survival and higher pathological grade of BCa patients. Therefore, SETD7 has great application potential as a diagnostic and therapeutic target in BCa. The mechanism network of SETD7 was shown in Figure 9.

## Supplementary Material

Supplementary figures and tables.

Supplementary table 1.

Supplementary table 2.

Supplementary table 3.

## Figures and Tables

**Table 1:** Correlations between the expression of SETD7 and clinicopathological features in BCa patients.

Characteristics	Case	SETD7		P value
		Low	High	
All cases	40	20	20	
Age(years)				0.2003
<65	17	6	11	
≥65	23	14	9	
Gender				0.9999
Male	31	15	16	
Female	9	5	4	
TNM stage				0.9999
pTa-pT1	9	5	4	
pT2-pT4	31	15	16	
Histological grade				0.0310^*^
Low	11	9	2	
High	29	11	18	
Tumor size(cm)				0.7411
<3 ≥3	14 26	8 12	6 14	
Lymph metastasis				0.5145
No yes	25 15	14 6	11 9	

*P<0.05

**Table 2:** Differentially expressed chemokines, chemokine receptors, MHC molecules, and immunostimulators in high and low SETD7 groups of GSE48075.

ID	logFC	AveExpression	P.Value
TGFB1	0.0940282	5.843602485	0.002173294
LAG3	-0.249996415	6.832321268	0.004344775
CXCL13	-0.339310765	6.371763716	0.012073098
KLRC1	-0.044108584	5.833786749	0.018697717
TNFRSF13B	0.109459043	6.034620352	0.028281531
CXCL5	0.313672048	6.179647894	0.033401792
CCR5	-0.045459596	5.736668054	0.037136087

**Table 3:** Differentially expressed immune cell (CD8^+^T cell, NK cell, Macrophage, Th1 cell, and DC cell) related effector genes in high and low SETD7 groups of GSE48075.

ID	logFC	AveExpression	P.Value
SETD7	0.314166374	5.936132122	1.40E-12
GZMA	-0.215659541	7.050527572	0.017162063
GZMB	-0.273299828	7.281140332	0.01872506
CD8B	-0.050217161	5.808694316	0.023834234
C1QA	0.141289967	7.144261055	0.047471261
FGL2	0.198225352	7.796804945	0.049420623

**Table 4:** Differentially expressed chemokines, chemokine receptors, MHC molecules, and immunostimulators in high and low SETD7 groups of TCGA BLCA.

ID	logFC	AveExpression	P.Value
TNFRSF4	-0.407137421	1.582464119	1.08E-12
TMIGD2	-0.445251976	0.795417781	1.32E-11
TNFRSF14	-0.715415009	3.356538579	2.83E-11
TNFRSF25	-0.613309814	2.627738865	5.86E-08
HLA-DRA	0.583715585	7.70825671	2.11E-07
CCL17	-0.538172924	1.638145542	7.09E-07
LGALS9	-0.569696603	3.597396054	7.71E-07
CCL15	-0.620342353	0.93922268	1.94E-06
CCR8	0.093095014	0.27206159	2.03E-06
CD96	-0.49576427	1.591052003	3.25E-06
CSF1R	0.359041141	2.442599956	7.21E-06
CCR10	-0.251528734	0.566202897	1.24E-05
XCL1	-0.4886411	1.409149168	2.75E-05
HLA-DPA1	0.394422327	4.114427063	5.91E-05
CCL16	-0.067221234	0.092207481	0.000109485
IL2RA	0.196031929	1.07875586	0.000127726
TAPBP	-0.302843855	6.070662282	0.000152004
CXCL12	0.3857595	1.92221197	0.000176002
PDCD1LG2	0.215815478	0.928031866	0.000232273
CCR4	0.118015139	0.470426063	0.000248861
LAG3	-0.251203848	1.375311732	0.000264299
HLA-G	-0.362427854	1.92341689	0.000270449
B2M	0.169294069	8.99417049	0.000374946
CCR7	-0.689555791	2.306014798	0.000435871
KLRK1	-0.115853533	0.286989921	0.000522171
CXCL6	0.422502434	1.226375287	0.000599695
HLA-E	-0.214757838	7.760561914	0.000662858
TNFSF15	-0.362577616	1.371837531	0.000815083
XCL2	-0.168158453	0.761013359	0.000866183
CCL24	0.254893487	0.706655772	0.000868957
HLA-C	-0.174434807	8.588157838	0.001000305
CXCL16	0.259851172	4.304914039	0.001451149
CXCL1	0.581825778	3.136808613	0.001482315
CCL18	0.489522021	2.695811792	0.002117743
HLA-DQA2	0.418881875	2.648273266	0.002249262
CD40	-0.350087943	3.205388013	0.002363755
CXCL8	0.526108396	3.222638289	0.002551165
CD86	0.154359192	1.600164547	0.002819366
IL6	0.343055321	1.4301635	0.003029053
CD28	0.068723151	0.439886638	0.00306783
HAVCR2	0.142531474	1.587647145	0.003370772
ADORA2A	-0.032075952	0.093291583	0.003392092
CXCL5	0.370108201	1.032458326	0.003793537
CCL21	0.516393881	2.267256929	0.004048087
HLA-F	-0.262768636	3.867934178	0.004771635
IL10RB	-0.267559224	4.214667998	0.008656195
HLA-DPB1	0.236591498	5.160928829	0.010752326
CCL11	0.32459946	2.019990598	0.010962466
LTA	-0.058556672	0.373856448	0.013888889
CCR5	0.098843923	0.994977925	0.01440415
CXCL9	0.377068183	2.727424704	0.01508146
TNFRSF13C	-0.172712801	0.868419458	0.016257377
HLA-DMA	-0.180918857	4.331179003	0.016307168
BTNL2	-0.055730648	0.086240691	0.019274478
NT5E	0.298436335	2.271548327	0.019336906
TNFRSF9	0.066804829	0.417889396	0.021310605
HLA-DQA1	0.217986422	2.856717162	0.02193436
CXCL14	-0.463599732	3.62531065	0.029183427
CCL25	-0.04165418	0.096789924	0.033265762
CCL27	-0.007559437	0.008955273	0.03501231
CCL1	-0.030209583	0.061494262	0.04091902
HLA-A	-0.132504947	8.448689213	0.043392131
CD160	-0.042727185	0.186458566	0.045786303

**Table 5:** Differentially expressed immune cell (CD8^+^T cell, NK cell, Macrophage, Th1 cell, and DC cell) related effector genes in high and low SETD7 groups of TCGA BLCA.

ID	logFC	AveExpression	P.Value
CYBB	0.277485941	2.279801429	9.53E-08
FLT3LG	-0.349522585	1.209018127	6.77E-07
GZMM	-0.223314051	1.057855356	2.12E-05
SLC15A3	-0.283485613	2.328144798	0.000204157
LILRB4	0.142925207	1.118869095	0.000280945
IL7R	0.238754074	1.578232597	0.000324303
CSF1R	0.220433422	2.491596023	0.000536239
SETD7	0.330445576	2.562436702	0.006119062
CTLA4	-0.105142566	1.013110816	0.007120342
MARCO	0.215894954	1.12246782	0.015978341
CCL5	-0.229649871	3.927848786	0.016375779
MMP8	0.027582645	0.085985423	0.020510011
CD8B	-0.167035087	1.033215369	0.028939501
C1QB	0.13254823	4.997479017	0.044915563

## Abbreviations

BCa: Bladder cancer
SETD7: SET Domain Containing 7
OS: Overall survival
DFS: Disease free survival
TME: Tumor microenvironment
BCa: Bladder cancer
NMIBC: Non-muscle invasive bladder cancer
MIBC: Muscle invasive bladder cancer
ICIs: Immune checkpoint inhibitors
PTMs: Post-translational modifications
HDACs: Histone deacetylases
NSCLC: Non-small-cell lung carcinoma
HPA: The Human Protein Atlas
qRT-PCR: Quantitative real-time PCR
CCK-8: Cell counting kit-8
CO-IP: Co-inmunoprecipitation
PBMCs: Peripheral blood mononuclear cells
Treg: T-regulatory cell
HuNOG: Human immune reconstitution NOG

## References

[1] Xia C, Dong X, Li H, Cao M, Sun D, He S (2022). Cancer statistics in China and United States, 2022: profiles, trends, and determinants. Chinese medical journal.

[2] Antoni S, Ferlay J, Soerjomataram I, Znaor A, Jemal A, Bray F (2017). Bladder Cancer Incidence and Mortality: A Global Overview and Recent Trends. Eur Urol.

[3] van Rhijn BW, Burger M, Lotan Y, Solsona E, Stief CG, Sylvester RJ (2009). Recurrence and progression of disease in non-muscle-invasive bladder cancer: from epidemiology to treatment strategy. Eur Urol.

[4] Tran L, Xiao JF, Agarwal N, Duex JE, Theodorescu D (2021). Advances in bladder cancer biology and therapy. Nature reviews Cancer.

[5] Lenis AT, Lec PM, Chamie K, Mshs MD (2020). Bladder Cancer: A Review. Jama.

[6] Patel VG, Oh WK, Galsky MD (2020). Treatment of muscle-invasive and advanced bladder cancer in 2020. CA Cancer J Clin.

[7] Balar AV, Galsky MD, Rosenberg JE, Powles T, Petrylak DP, Bellmunt J (2017). Atezolizumab as first-line treatment in cisplatin-ineligible patients with locally advanced and metastatic urothelial carcinoma: a single-arm, multicentre, phase 2 trial. Lancet (London, England).

[8] Strahl BD, Allis CD (2000). The language of covalent histone modifications. Nature.

[9] Berger SL (2002). Histone modifications in transcriptional regulation. Current opinion in genetics & development.

[10] Quivy V, Calomme C, Dekoninck A, Demonte D, Bex F, Lamsoul I (2004). Gene activation and gene silencing: a subtle equilibrium. Cloning and stem cells.

[11] Wang S, Osgood AO, Chatterjee A (2022). Uncovering post-translational modification-associated protein-protein interactions. Current opinion in structural biology.

[12] Li W, Li F, Zhang X, Lin HK, Xu C (2021). Insights into the post-translational modification and its emerging role in shaping the tumor microenvironment. Signal transduction and targeted therapy.

[13] Chen L, Liu S, Tao Y (2020). Regulating tumor suppressor genes: post-translational modifications. Signal transduction and targeted therapy.

[14] Gil J, Ramírez-Torres A, Encarnación-Guevara S (2017). Lysine acetylation and cancer: A proteomics perspective. Journal of proteomics.

[15] Swatek KN, Komander D (2016). Ubiquitin modifications. Cell research.

[16] Horvat S, Roscić M (2010). Glycosylation of lysine-containing pentapeptides by glucuronic acid: new insights into the Maillard reaction. Carbohydrate research.

[17] Carlson SM, Gozani O (2016). Nonhistone Lysine Methylation in the Regulation of Cancer Pathways. Cold Spring Harbor perspectives in medicine.

[18] Wan J, Liu H, Chu J, Zhang H (2019). Functions and mechanisms of lysine crotonylation. Journal of cellular and molecular medicine.

[19] Lanouette S, Mongeon V, Figeys D, Couture JF (2014). The functional diversity of protein lysine methylation. Molecular systems biology.

[20] Han D, Huang M, Wang T, Li Z, Chen Y, Liu C (2019). Lysine methylation of transcription factors in cancer. Cell death & disease.

[21] Wang G, Long J, Gao Y, Zhang W, Han F, Xu C (2019). SETDB1-mediated methylation of Akt promotes its K63-linked ubiquitination and activation leading to tumorigenesis. Nature cell biology.

[22] Park SC, Lee JM (2022). Ezh2 promotes TRβ lysine methylation-mediated degradation in hepatocellular carcinoma. Genes & genomics.

[23] Xiao G, Jin LL, Liu CQ, Wang YC, Meng YM, Zhou ZG (2019). EZH2 negatively regulates PD-L1 expression in hepatocellular carcinoma. Journal for immunotherapy of cancer.

[24] Bugide S, Gupta R, Green MR, Wajapeyee N (2021). EZH2 inhibits NK cell-mediated antitumor immunity by suppressing CXCL10 expression in an HDAC10-dependent manner. Proceedings of the National Academy of Sciences of the United States of America.

[25] Fick RJ, Kroner GM, Nepal B, Magnani R, Horowitz S, Houtz RL (2016). Sulfur-Oxygen Chalcogen Bonding Mediates AdoMet Recognition in the Lysine Methyltransferase SET7/9. ACS chemical biology.

[26] Nishioka K, Chuikov S, Sarma K, Erdjument-Bromage H, Allis CD, Tempst P (2002). Set9, a novel histone H3 methyltransferase that facilitates transcription by precluding histone tail modifications required for heterochromatin formation. Genes & development.

[27] Dhayalan A, Kudithipudi S, Rathert P, Jeltsch A (2011). Specificity analysis-based identification of new methylation targets of the SET7/9 protein lysine methyltransferase. Chemistry & biology.

[28] Chiang C, Yang H, Zhu L, Chen C, Chen C, Zuo Y (2022). The Epigenetic Regulation of Nonhistone Proteins by SETD7: New Targets in Cancer. Front Genet.

[29] Fu L, Wu H, Cheng SY, Gao D, Zhang L, Zhao Y (2016). Set7 mediated Gli3 methylation plays a positive role in the activation of Sonic Hedgehog pathway in mammals. eLife.

[30] Cao L, Ren Y, Guo X, Wang L, Zhang Q, Li X (2020). Downregulation of SETD7 promotes migration and invasion of lung cancer cells via JAK2/STAT3 pathway. International journal of molecular medicine.

[31] Xie H, Li J, Ying Y, Yan H, Jin K, Ma X (2020). METTL3/YTHDF2 m(6) A axis promotes tumorigenesis by degrading SETD7 and KLF4 mRNAs in bladder cancer. Journal of cellular and molecular medicine.

[32] Liu C, Yao Z, Wang J, Zhang W, Yang Y, Zhang Y (2020). Macrophage-derived CCL5 facilitates immune escape of colorectal cancer cells via the p65/STAT3-CSN5-PD-L1 pathway. Cell death and differentiation.

[33] Song TL, Nairismägi ML, Laurensia Y, Lim JQ, Tan J, Li ZM (2018). Oncogenic activation of the STAT3 pathway drives PD-L1 expression in natural killer/T-cell lymphoma. Blood.

[34] Huseni MA, Wang L, Klementowicz JE, Yuen K, Breart B, Orr C (2023). CD8(+) T cell-intrinsic IL-6 signaling promotes resistance to anti-PD-L1 immunotherapy. Cell reports Medicine.

[35] Lv J, Li K, Yu H, Han J, Zhuang J, Yu R (2023). HNRNPL induced circFAM13B increased bladder cancer immunotherapy sensitivity via inhibiting glycolysis through IGF2BP1/PKM2 pathway. Journal of experimental & clinical cancer research: CR.

[36] de Brevern AG, Rebehmed J (2022). Current status of PTMs structural databases: applications, limitations and prospects. Amino acids.

[37] Pan S, Chen R (2022). Pathological implication of protein post-translational modifications in cancer. Molecular aspects of medicine.

[38] Campbell SL, Philips MR (2021). Post-translational modification of RAS proteins. Current opinion in structural biology.

[39] Wang K, Liu J, Li YL, Li JP, Zhang R (2022). Ubiquitination/de-ubiquitination: A promising therapeutic target for PTEN reactivation in cancer. Biochimica et biophysica acta Reviews on cancer.

[40] Cordier F, Chaffotte A, Wolff N (2015). Quantitative and dynamic analysis of PTEN phosphorylation by NMR. Methods (San Diego, Calif).

[41] Egorova KS, Olenkina OM, Olenina LV (2010). Lysine methylation of nonhistone proteins is a way to regulate their stability and function. Biochemistry Biokhimiia.

[42] Wang Q, Wang K, Ye M (2017). Strategies for large-scale analysis of non-histone protein methylation by LC-MS/MS. The Analyst.

[43] Zhang Q, Liu S, Wang H, Xiao K, Lu J, Chen S (2023). ETV4 Mediated Tumor-Associated Neutrophil Infiltration Facilitates Lymphangiogenesis and Lymphatic Metastasis of Bladder Cancer. Advanced science (Weinheim, Baden-Wurttemberg, Germany).

[44] Xie R, Cheng L, Huang M, Huang L, Chen Z, Zhang Q (2023). NAT10 Drives Cisplatin Chemoresistance by Enhancing ac4C-Associated DNA Repair in Bladder Cancer. Cancer Res.

[45] Elkouris M, Kontaki H, Stavropoulos A, Antonoglou A, Nikolaou KC, Samiotaki M (2016). SET9-Mediated Regulation of TGF-β Signaling Links Protein Methylation to Pulmonary Fibrosis. Cell reports.

[46] Oudhoff MJ, Braam MJS, Freeman SA, Wong D, Rattray DG, Wang J (2016). SETD7 Controls Intestinal Regeneration and Tumorigenesis by Regulating Wnt/β-Catenin and Hippo/YAP Signaling. Developmental cell.

[47] Gaughan L, Stockley J, Wang N, McCracken SR, Treumann A, Armstrong K (2011). Regulation of the androgen receptor by SET9-mediated methylation. Nucleic acids research.

[48] Kim Y, Nam HJ, Lee J, Park DY, Kim C, Yu YS (2016). Methylation-dependent regulation of HIF-1α stability restricts retinal and tumour angiogenesis. Nature communications.

[49] Han T, Wan Y, Wang J, Zhao P, Yuan Y, Wang L (2015). Set7 facilitates hepatitis C virus replication via enzymatic activity-dependent attenuation of the IFN-related pathway. Journal of immunology (Baltimore, Md: 1950).

[50] Wu R, Wu X, Zou L, Zhou L, Mao Y (2023). DDB1 regulates the activation-induced apoptosis of T cells via downregulating the expression of histone methyltransferase SETD7. Medical oncology (Northwood, London, England).

[51] Zhang W, Zhang J, Zhang Z, Guo Y, Wu Y, Wang R (2019). Overexpression of Indoleamine 2,3-Dioxygenase 1 Promotes Epithelial-Mesenchymal Transition by Activation of the IL-6/STAT3/PD-L1 Pathway in Bladder Cancer. Translational oncology.

[52] Hays E, Bonavida B (2019). YY1 regulates cancer cell immune resistance by modulating PD-L1 expression. Drug resistance updates: reviews and commentaries in antimicrobial and anticancer chemotherapy.

[53] Cao D, Qi Z, Pang Y, Li H, Xie H, Wu J (2019). Retinoic Acid-Related Orphan Receptor C Regulates Proliferation, Glycolysis, and Chemoresistance via the PD-L1/ITGB6/STAT3 Signaling Axis in Bladder Cancer. Cancer Res.

