# Supplementary material for: Lysine N-methyltransferase SETD7 promotes bladder cancer progression and immune escape via STAT3/PD-L1 cascade
Source: Int J Biol Sci. 2023 Jul 16;19(12):3744–61. doi: 10.7150/ijbs.87182 (PMC10411476; doi:10.7150/ijbs.87182)
Supplement: Supplementary file 1 — Supplementary figures and tables. [file ijbsv19p3744s1.pdf]

Figure S1: Prognostic value of the lysine methytransferase family in patients with BCa.
A-M: The overall survival and disease-free survival outcomes of 13 lysine methytransferase in TCGA-BLCA data (SETDB1, METTL21A, SETD1A, SETD8, NSD1, EHMT2, SMYD3, EZH2, METTL22, SMYD2, SETD6, CAMKMT, SETD7).

Figure S2: Correlation between lysine methytransferase family and immunotherapy response of patients with BCa.
A-L: Correlations between 12 lysine methytransferase and immunotherapy response of patients with BCa in GSE173839 (SETDB1, METTL21A, SETD1A, NSD1, EHMT2, SMYD3, EZH2, METTL22, SMYD2, SETD6, CAMKMT, and SETD7).

Figure S3: SETD7 was negatively correlated with the BCa immunescore of GSE69795.
A. Infiltration level of different immune cells in high- and low-SETD7 groups on the basis of GSE69795 data. **B.** Correlation analysis of SETD7 and immunescore which was based on the immune cell infiltration level of GSE69795 data. **C.** Expression of different immunomodulators in high- and low-SETD7 groups on the basis of GSE69795 data. **D.** Expression of immune cells effector genes in high and low SETD7 groups on the basis of GSE69795 data. **E.** Correlation analysis between SETD7 and inhibitory immune checkpoints on the basis of GSE69795 data.

Figure S4: Associations between SETD7 and immune cells infiltration in TCGA BLCA.

Figure S5: The GO-GSEA and Hallmark-GSEA analyses of SETD7 in BCa.

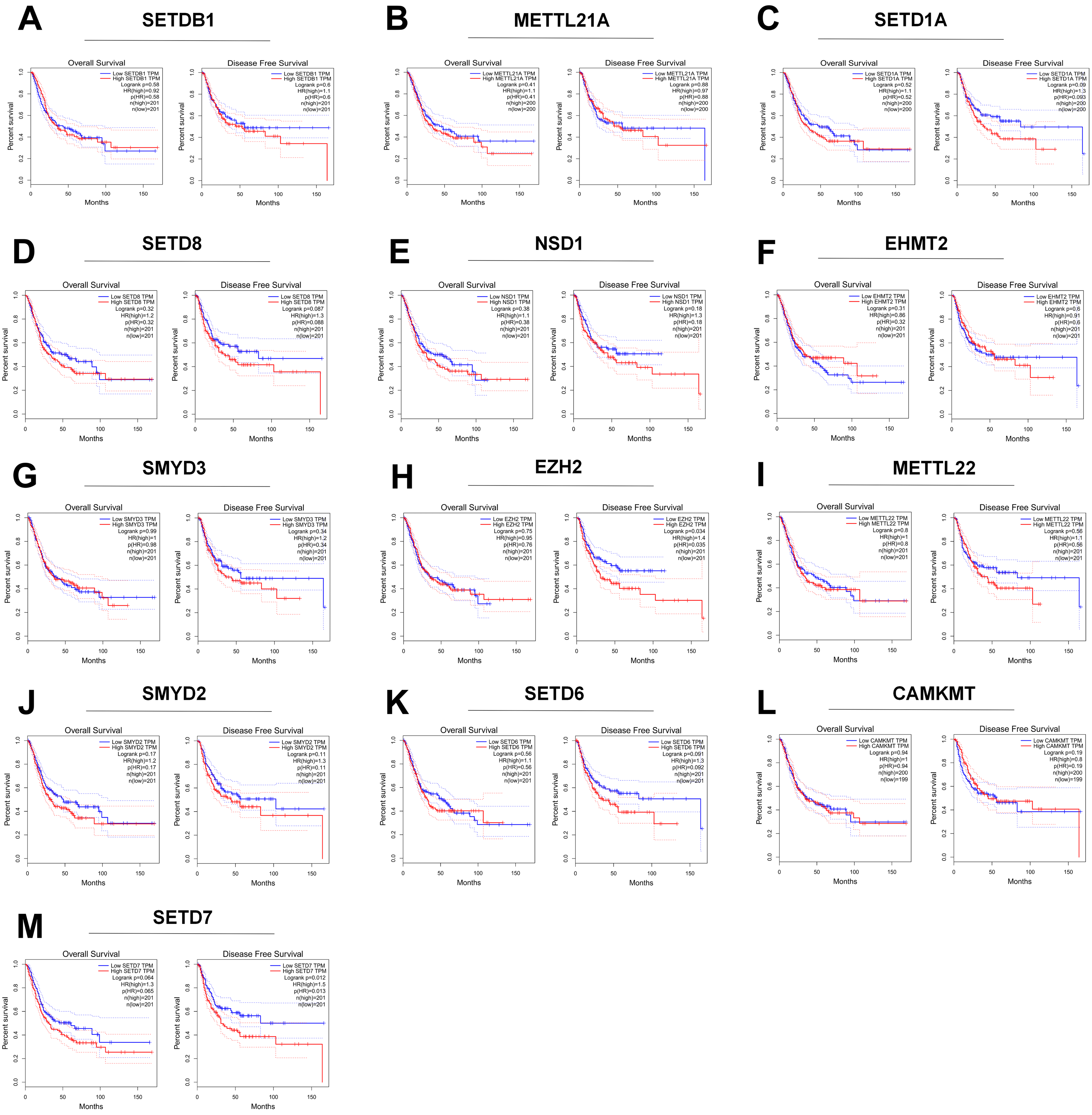
A
SETDB1
Overall Survival
Low SETDB1 TPM
High SETDB1 TPM
Logrank p=0.58
HR(high)=0.92
p(HR)=0.58
n(high)=201
n(low)=201
Disease Free Survival
Low SETDB1 TPM
High SETDB1 TPM
Logrank p=0.6
HR(high)=1.1
p(HR)=0.6
n(high)=201
n(low)=201
B
METTL21A
Overall Survival
Low METTL21A TPM
High METTL21A TPM
Logrank p=0.41
HR(high)=1.1
p(HR)=0.41
n(high)=200
n(low)=200
Disease Free Survival
Low METTL21A TPM
High METTL21A TPM
Logrank p=0.88
HR(high)=0.97
p(HR)=0.88
n(high)=200
n(low)=200
C
SETD1A
Overall Survival
Low SETD1A TPM
High SETD1A TPM
Logrank p=0.52
HR(high)=1.1
p(HR)=0.52
n(high)=200
n(low)=200
Disease Free Survival
Low SETD1A TPM
High SETD1A TPM
Logrank p=0.09
HR(high)=1.3
p(HR)=0.093
n(high)=200
n(low)=200
D
SETD8
Overall Survival
Low SETD8 TPM
High SETD8 TPM
Logrank p=0.32
HR(high)=1.2
p(HR)=0.32
n(high)=201
n(low)=201
Disease Free Survival
Low SETD8 TPM
High SETD8 TPM
Logrank p=0.087
HR(high)=1.3
p(HR)=0.088
n(high)=201
n(low)=201
E
NSD1
Overall Survival
Low NSD1 TPM
High NSD1 TPM
Logrank p=0.38
HR(high)=1.1
p(HR)=0.38
n(high)=201
n(low)=201
Disease Free Survival
Low NSD1 TPM
High NSD1 TPM
Logrank p=0.18
HR(high)=1.3
p(HR)=0.18
n(high)=201
n(low)=201
F
EHMT2
Overall Survival
Low EHMT2 TPM
High EHMT2 TPM
Logrank p=0.31
HR(high)=0.86
p(HR)=0.32
n(high)=201
n(low)=201
Disease Free Survival
Low EHMT2 TPM
High EHMT2 TPM
Logrank p=0.6
HR(high)=0.91
p(HR)=0.6
n(high)=201
n(low)=201
G
SMYD3
Overall Survival
Low SMYD3 TPM
High SMYD3 TPM
Logrank p=0.99
HR(high)=1
p(HR)=0.98
n(high)=201
n(low)=201
Disease Free Survival
Low SMYD3 TPM
High SMYD3 TPM
Logrank p=0.34
HR(high)=1.2
p(HR)=0.34
n(high)=201
n(low)=201
H
EZH2
Overall Survival
Low EZH2 TPM
High EZH2 TPM
Logrank p=0.75
HR(high)=0.95
p(HR)=0.76
n(high)=201
n(low)=201
Disease Free Survival
Low EZH2 TPM
High EZH2 TPM
Logrank p=0.034
HR(high)=1.4
p(HR)=0.035
n(high)=201
n(low)=201
I
METTL22
Overall Survival
Low METTL22 TPM
High METTL22 TPM
Logrank p=0.8
HR(high)=1
p(HR)=0.8
n(high)=201
n(low)=201
Disease Free Survival
Low METTL22 TPM
High METTL22 TPM
Logrank p=0.56
HR(high)=1.1
p(HR)=0.56
n(high)=201
n(low)=201
J
SMYD2
Overall Survival
Low SMYD2 TPM
High SMYD2 TPM
Logrank p=0.17
HR(high)=1.2
p(HR)=0.17
n(high)=201
n(low)=201
Disease Free Survival
Low SMYD2 TPM
High SMYD2 TPM
Logrank p=0.11
HR(high)=1.3
p(HR)=0.11
n(high)=201
n(low)=201
K
SETD6
Overall Survival
Low SETD6 TPM
High SETD6 TPM
Logrank p=0.56
HR(high)=1.1
p(HR)=0.56
n(high)=201
n(low)=201
Disease Free Survival
Low SETD6 TPM
High SETD6 TPM
Logrank p=0.091
HR(high)=1.3
p(HR)=0.092
n(high)=201
n(low)=201
L
CAMKMT
Overall Survival
Low CAMKMT TPM
High CAMKMT TPM
Logrank p=0.94
HR(high)=1
p(HR)=0.94
n(high)=200
n(low)=199
Disease Free Survival
Low CAMKMT TPM
High CAMKMT TPM
Logrank p=0.19
HR(high)=0.8
p(HR)=0.19
n(high)=200
n(low)=199
M
SETD7
Overall Survival
Low SETD7 TPM
High SETD7 TPM
Logrank p=0.064
HR(high)=1.3
p(HR)=0.065
n(high)=201
n(low)=201
Disease Free Survival
Low SETD7 TPM
High SETD7 TPM
Logrank p=0.012
HR(high)=1.5
p(HR)=0.013
n(high)=201
n(low)=201
Percent survival
Months

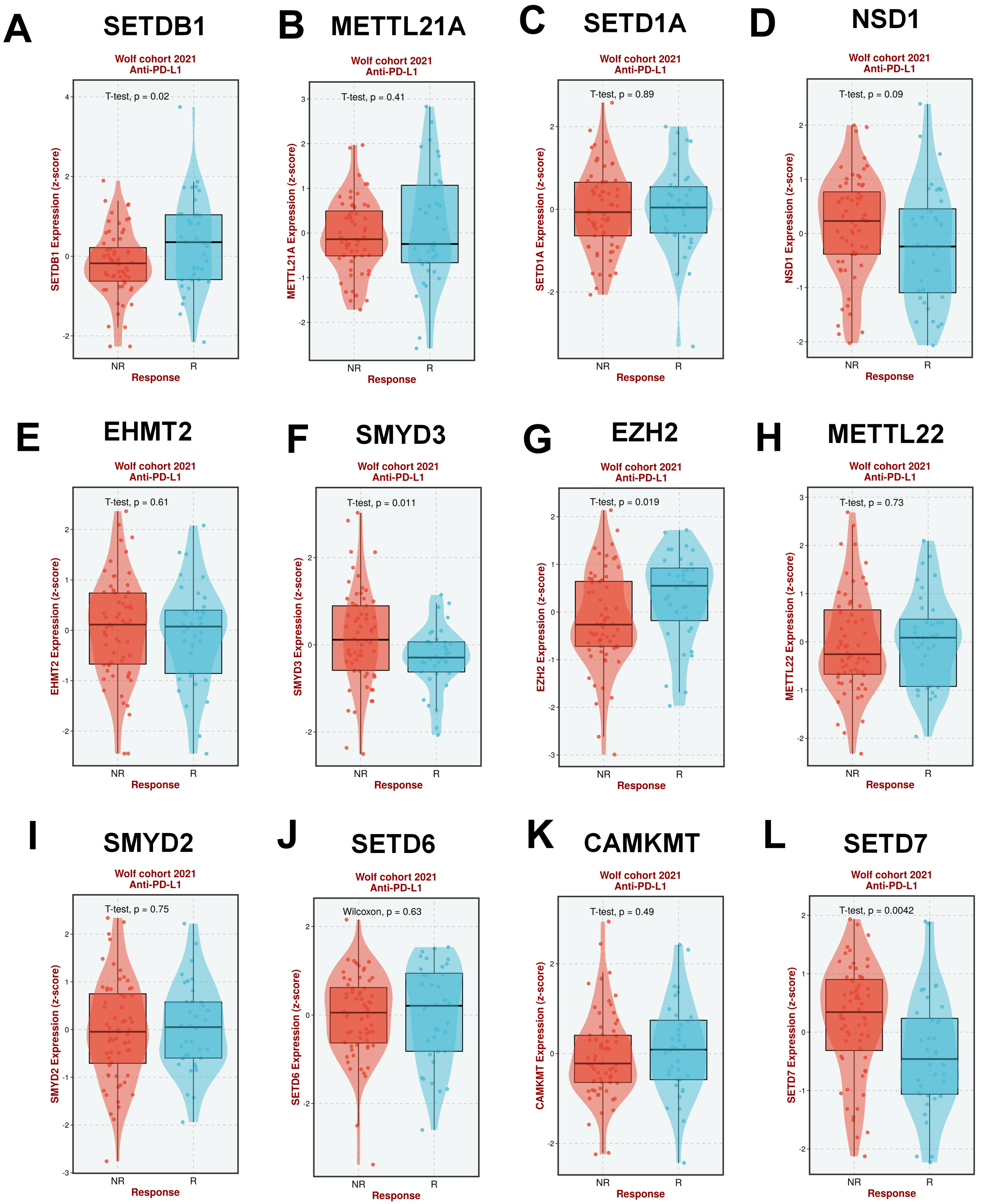

A
SETDB1
Wolf cohort 2021
Anti-PD-L1
T-test, p = 0.02
SETDB1 Expression (z-score)
NR
R
Response
B
METTL21A
Wolf cohort 2021
Anti-PD-L1
T-test, p = 0.41
METTL21A Expression (z-score)
NR
R
Response
C
SETD1A
Wolf cohort 2021
Anti-PD-L1
T-test, p = 0.89
SETD1A Expression (z-score)
NR
R
Response
D
NSD1
Wolf cohort 2021
Anti-PD-L1
T-test, p = 0.09
NSD1 Expression (z-score)
NR
R
Response
E
EHMT2
Wolf cohort 2021
Anti-PD-L1
T-test, p = 0.61
EHMT2 Expression (z-score)
NR
R
Response
F
SMYD3
Wolf cohort 2021
Anti-PD-L1
T-test, p = 0.011
SMYD3 Expression (z-score)
NR
R
Response
G
EZH2
Wolf cohort 2021
Anti-PD-L1
T-test, p = 0.019
EZH2 Expression (z-score)
NR
R
Response
H
METTL22
Wolf cohort 2021
Anti-PD-L1
T-test, p = 0.73
METTL22 Expression (z-score)
NR
R
Response
I
SMYD2
Wolf cohort 2021
Anti-PD-L1
T-test, p = 0.75
SMYD2 Expression (z-score)
NR
R
Response
J
SETD6
Wolf cohort 2021
Anti-PD-L1
Wilcoxon, p = 0.63
SETD6 Expression (z-score)
NR
R
Response
K
CAMKMT
Wolf cohort 2021
Anti-PD-L1
T-test, p = 0.49
CAMKMT Expression (z-score)
NR
R
Response
L
SETD7
Wolf cohort 2021
Anti-PD-L1
T-test, p = 0.0042
SETD7 Expression (z-score)
NR
R
Response

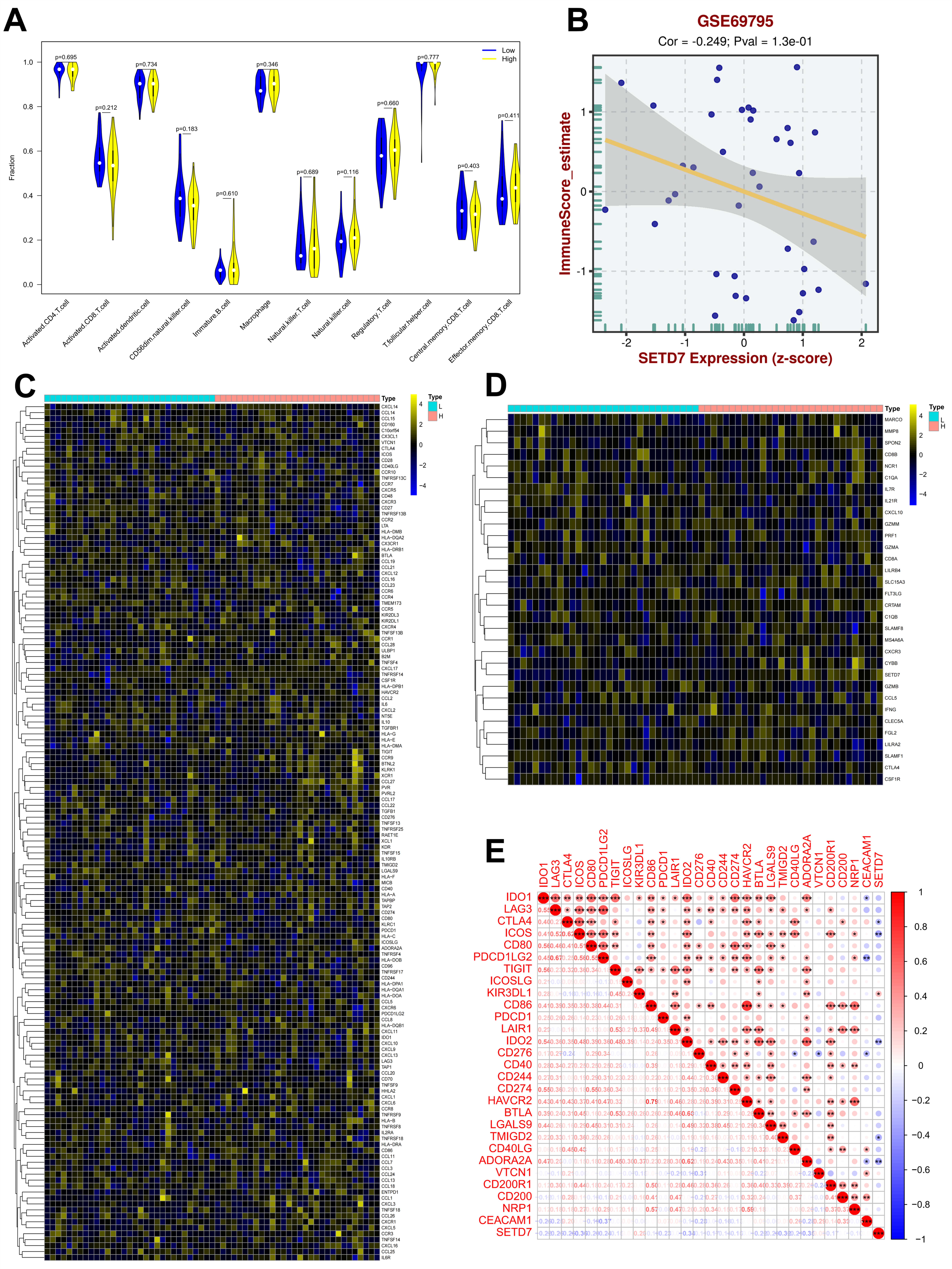
A
Fraction
Low
High
p=0.695
p=0.212
p=0.734
p=0.183
p=0.610
p=0.346
p=0.689
p=0.116
p=0.660
p=0.777
p=0.403
p=0.411
Activated.CD4.T.cell
Activated.CD8.T.cell
Activated.dendritic.cell
CD56dim.natural.killer.cell
Immature.B.cell
Macrophage
Natural.killer.T.cell
Natural.killer.cell
Regulatory.T.cell
T.follicular.helper.cell
Central.memory.CD8.T.cell
Effector.memory.CD8.T.cell
B
GSE69795
Cor = -0.249; Pval = 1.3e-01
ImmuneScore_estimate
SETD7 Expression (z-score)
C
Type
L
H
CXCL14 CCL14 CCL15 C10orf54 CX3CL1 VTCN1 CTLA4 ICOS CD28 CD40LG CCR10 TNFRSF13C CCR7 CXCR5 CD48 CXCR3 CD27 TNFRSF13B CCR2 LTA HLA-DMB HLA-DQA2 CX3CR1 HLA-DRB1 BTLA CCL19 CCL21 CXCL12 CCL16 CCL23 CCR6 CCR4 TMEM173 CCR5 KIR2DL3 KIR2DL1 CXCR4 TNFSF13B CCR1 CCL28 ULBP1 B2M TNFSF4 CXCL17 TNFRSF14 CSF1R HLA-DPB1 HAVCR2 CCL2 IL6 CXCL2 NT5E IL10 TGFBR1 HLA-G HLA-E HLA-DMA TIGIT CCR9 BTNL2 KLRK1 XCR1 CCL27 PVR PVRL2 CCL17 CCL22 TGFB1 CD276 TNFSF13 TNFRSF25 RAET1E XCL1 KDR TNFSF15 IL10RB TMIGD2 LGALS9 HLA-F MICB CD40 HLA-A TAPBP TAP2 CD274 CD80 KLRC1 PDCD1 HLA-C ICOSLG ADORA2A TNFRSF4 HLA-DOB CD96 TNFRSF17 CD244 HLA-DPA1 HLA-DQA1 HLA-DOA CCL5 CXCR6 PDCD1LG2 CCL8 HLA-DQB1 CXCL11 IDO1 CXCL10 CXCL9 CXCL13 LAG3 TAP1 CCL20 CD70 TNFSF9 HHLA2 CXCL1 CXCL6 CCR8 TNFRSF9 HLA-B TNFRSF8 IL2RA TNFRSF18 HLA-DRA CD86 CCL11 CCL7 CCL3 CCL24 CCL13 CCL18 ENTPD1 CCL1 CXCL3 TNFSF18 CCL26 CXCR1 CXCL5 CCR3 TNFSF14 CXCL16 CCL25 IL6R
D
Type
L
H
MARCO MMP8 SPON2 CD8B NCR1 C1QA IL7R IL21R CXCL10 GZMM PRF1 GZMA CD8A LILRB4 SLC15A3 FLT3LG CRTAM C1QB SLAMF8 MS4A6A CXCR3 CYBB SETD7 GZMB CCL5 IFNG CLEC5A FGL2 LILRA2 SLAMF1 CTLA4 CSF1R
E
IDO1 LAG3 CTLA4 ICOS CD80 PDCD1LG2 TIGIT ICOSLG KIR3DL1 CD86 PDCD1 LAIR1 IDO2 CD276 CD40 CD244 CD274 HAVCR2 BTLA LGALS9 TMIGD2 CD40LG ADORA2A VTCN1 CD200R1 CD200 NRP1 CEACAM1 SETD7

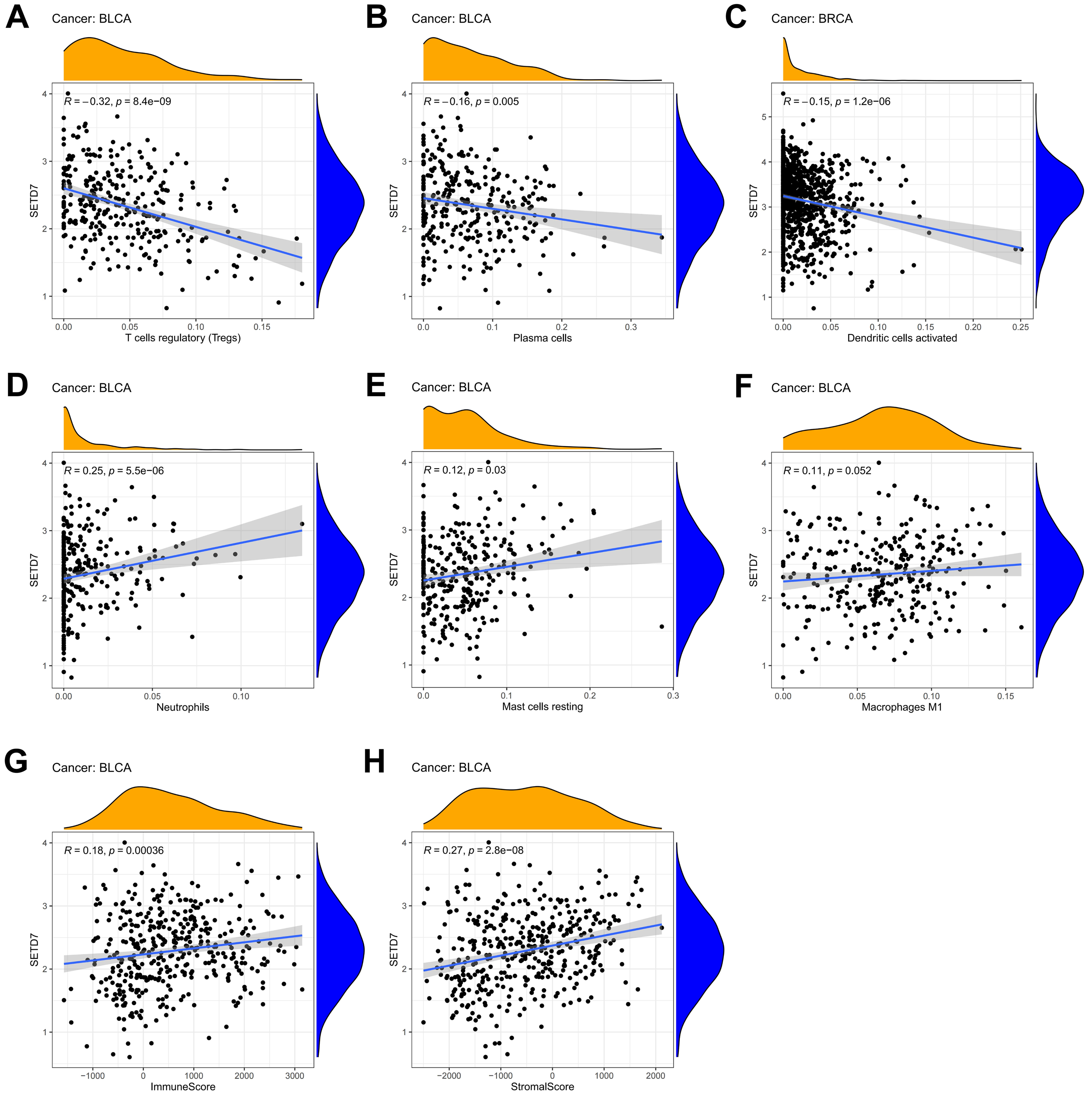
A
Cancer: BLCA
R = −0.32, p = 8.4e−09
SETD7
T cells regulatory (Tregs)
B
Cancer: BLCA
R = −0.16, p = 0.005
SETD7
Plasma cells
C
Cancer: BRCA
R = −0.15, p = 1.2e−06
SETD7
Dendritic cells activated
D
Cancer: BLCA
R = 0.25, p = 5.5e−06
SETD7
Neutrophils
E
Cancer: BLCA
R = 0.12, p = 0.03
SETD7
Mast cells resting
F
Cancer: BLCA
R = 0.11, p = 0.052
SETD7
Macrophages M1
G
Cancer: BLCA
R = 0.18, p = 0.00036
SETD7
ImmuneScore
H
Cancer: BLCA
R = 0.27, p = 2.8e−08
SETD7
StromalScore

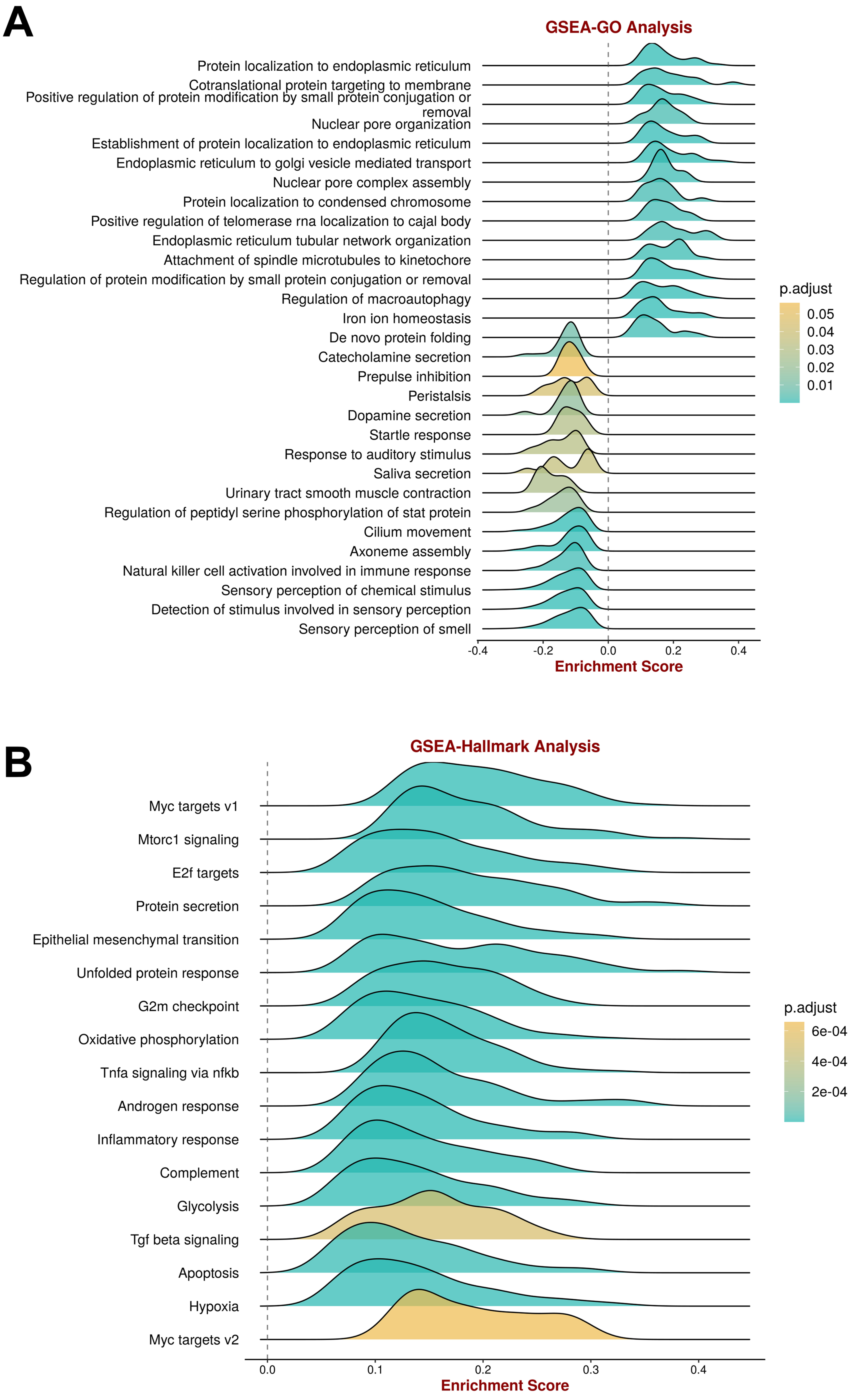
A
GSEA-GO Analysis
Protein localization to endoplasmic reticulum
Cotranslational protein targeting to membrane
Positive regulation of protein modification by small protein conjugation or removal
Nuclear pore organization
Establishment of protein localization to endoplasmic reticulum
Endoplasmic reticulum to golgi vesicle mediated transport
Nuclear pore complex assembly
Protein localization to condensed chromosome
Positive regulation of telomerase rna localization to cajal body
Endoplasmic reticulum tubular network organization
Attachment of spindle microtubules to kinetochore
Regulation of protein modification by small protein conjugation or removal
Regulation of macroautophagy
Iron ion homeostasis
De novo protein folding
Catecholamine secretion
Prepulse inhibition
Peristalsis
Dopamine secretion
Startle response
Response to auditory stimulus
Saliva secretion
Urinary tract smooth muscle contraction
Regulation of peptidyl serine phosphorylation of stat protein
Cilium movement
Axoneme assembly
Natural killer cell activation involved in immune response
Sensory perception of chemical stimulus
Detection of stimulus involved in sensory perception
Sensory perception of smell
-0.4
-0.2
0.0
0.2
0.4
Enrichment Score
p.adjust
0.05
0.04
0.03
0.02
0.01
B
GSEA-Hallmark Analysis
Myc targets v1
Mtorc1 signaling
E2f targets
Protein secretion
Epithelial mesenchymal transition
Unfolded protein response
G2m checkpoint
Oxidative phosphorylation
Tnfa signaling via nfkb
Androgen response
Inflammatory response
Complement
Glycolysis
Tgf beta signaling
Apoptosis
Hypoxia
Myc targets v2
0.0
0.1
0.2
0.3
0.4
Enrichment Score
p.adjust
6e-04
4e-04
2e-04

Table S4: All PCR primers used in this research

**Primers**		**Sequence （5'-3'）**
SETD7	Forward	5'-ATGGATAGCGACGACGAGATG-3'
	Reverse	5'-GCAGAACCCGTGCGGTAAT-3'
PD-L1	Forward	5'-TGGCATTTGCTGAACGCATTT-3'
	Reverse	5'-TGCAGCCAGGTCTAATTGTTTT-3'
β-Actin	Forward	5'-CTCCATCCTGGCCTCGCTGT-3'
	Reverse	5'-GCTGTCACCTTCACCGTTCC-3'

Table S5: Differentially expressed chemokines, chemokine receptors, MHC molecules, and immunostimulators in high and low SETD7 groups of GSE69795.

ID	logFC	AveExpression	P.Value
CD27	-1.05843677	10.68337821	0.000476409
KDR	0.921726243	10.94967268	0.001037254
CCL27	1.076677996	6.888451478	0.003797654
CCR9	0.371974514	5.96194796	0.005139314
CXCL17	0.922135828	13.56727185	0.005310415
TNFRSF17	-1.113525306	7.600444778	0.005756879
TNFRSF13B	-0.837040918	11.36619321	0.009039589
CX3CR1	0.861538602	8.406087679	0.010659964
KLRK1	0.461059444	7.095738245	0.015614535
CCR6	-0.357586301	12.50209037	0.026900344
HHLA2	0.283602898	5.447229856	0.033298687
CXCR6	-0.66946613	10.52301643	0.047939565
CCR2	0.498545176	7.366932452	0.049446249

Table S6: Differentially expressed immune cell (CD8$^+$T cell, NK cell, Macrophage, Th1 cell, and DC cell) related effector genes in high and low SETD7 groups of GSE69795.

ID	logFC	AveExpression	P.Value
SETD7	1.090535255	11.05232836	9.542E-06
GZMB	-0.468495417	12.54022642	0.019992446
GZMA	-0.481960831	12.19780344	0.024910009
SLAMF1	-0.701155072	8.632630496	0.03135724
